# Mapping the structural dynamics of red- and blue-emitting beetle luciferases revealed by HDX-MS

**DOI:** 10.26508/lsa.202503385

**Published:** 2025-10-23

**Authors:** Abdul-Rahman Kharbatli, Juliana C Ferreira, Nathan M Lui, Rawdah Karwt, Shaolong Zhu, Liaqat Ali, Wael M Rabeh

**Affiliations:** 1 Science Division, New York University Abu Dhabi , Abu Dhabi, United Arab Emirates; 2 Department of Genetics, Kazan Federal University , Kazan, Russia; 3 Department of Chemistry, York University , Toronto, Canada; 4 Core Technology Platforms, New York University Abu Dhabi , Saadiyat Campus, Abu Dhabi, United Arab Emirates

## Abstract

Beetle luciferases catalyze bioluminescent reactions using identical substrates—luciferin, ATP, and oxygen—yet emit colors ranging from green to red. As the luciferase fold is highly conserved, the structural basis of this spectral diversity remains elusive. Hydrogen/deuterium exchange mass spectrometry (HDX-MS) was used to systematically probe the conformational dynamics of green-emitting *Amydetes vivianii* luciferase (GB_Av_) and red-emitting *Phrixothrix hirtus* luciferase (RE_Ph_), along with their color-shifting mutants, R337L and L334R, respectively. HDX-MS profiling in the apo and ligand-bound states mapped dynamic changes associated with substrate binding and color modulation. RE_Ph_ and the red-shifted R337L GB_Av_ mutant exhibited globally higher dynamics, particularly in N-terminal domain peptides surrounding the active site, than their blue-green-emitting counterparts. Substrate binding reduced deuterium uptake in all enzymes; however, red-emitting forms retained greater flexibility. These findings establish a direct link between structural dynamics and bioluminescence color tuning in beetle luciferases, providing mechanistic insight into spectral control.

## Abstract (precis)

Using HDX-MS, this study demonstrates that conformational flexibility in beetle luciferases governs bioluminescence color, revealing how active site dynamics and domain-specific mutations fine-tune red- and blue-shifted light emission.

## Introduction

The enchanting glow of fireflies has intrigued both the scientific community and the general public for generations. Beyond its visual appeal, beetle bioluminescence has found significant utility in diverse fields such as in vivo imaging, cell tracking, protein folding studies, environmental monitoring, and food quality control. Central to this biological spectacle is the intricate process of energy transformation executed by the enzyme luciferase. In the presence of ATP and oxygen, luciferase catalyzes the conversion of chemical energy from a substrate in its ground state, luciferin, to the excited state through a spin-forbidden chemical reaction (Johnson & Shimomura, 1972; Deluca & Mcelroy, 1974).

Despite utilizing the same substrates, beetle luciferases from different species emit light across a spectrum of colors, from green–yellow to orange to red (Deluca & Mcelroy, 1974; Viviani & Bechara, 1995; Viviani et al, 1999b, 2001, 2002, 2004, 2005, 2006, 2011; Viviani & Ohmiya, 2000; Ugarova & Brovko, 2002; Viviani, 2002; Branchini et al, 2004a, 2004b, 2005a, 2005b, 2010, 2017; Ugarova et al, 2005; Li et al, 2010; Hirano et al, 2012; Amaral et al, 2016). The variations in light color are species-specific, with fireflies typically emitting yellow–green light, click beetles emitting light ranging from green to orange, and railroad worms displaying green or red light (Viviani et al, 1999a; 2011; Amaral et al, 2016; Branchini et al, 2017). These colors can also be altered through point mutations (Kajiyama & Nakano, 1991; Ugarova & Brovko, 2001, 2002; Branchini et al, 2004a, 2005a; Law et al, 2006; Reger et al, 2007; Tafreshi et al, 2007, 2008; Rand et al, 2008; Viviani et al, 2008, 2016; Edelheit et al, 2009; Iacob et al, 2009; Moradi et al, 2009; Emsley et al, 2010; Maghami et al, 2010; Koksharov & Ugarova, 2011a, 2011b, 2013; Wang et al, 2013; Modestova et al, 2014; Nishiguchi et al, 2015; Modestova & Ugarova, 2016). The molecular mechanisms responsible for the diverse color emissions of beetle luciferases have been a topic of ongoing scientific inquiry and debate since the 1970s, but remain elusive, despite extensive spectroscopic research and computational studies (Orlova et al, 2003; Hirano et al, 2009; Pinto da Silva & Esteves da Silva, 2012; Pinto da Silva et al, 2012; Yu & Liu, 2020). The multifaceted nature of bioluminescence, which involves bond formation and breakage, chemiexcitation, and emission processes, adds to the challenge.

The twisted intramolecular charge transfer (TICT) hypothesis, which posits that torsional rotation between oxyluciferin fragments leads to spectral shifts, has been largely discredited. Multiple computational and structural studies have consistently demonstrated that this mechanism fails to explain the observed emission properties of beetle luciferases (McCapra, 1994; Ugarova & Brovko, 2002; Nakatsu et al, 2006; Nakatani et al, 2007; Hirano et al, 2012). Other theories suggest that color differences arise from the influence of luciferase conformation and microenvironment on the electronic state of oxyluciferin (Moradi et al, 2009; Støchkel et al, 2013; Garcia-Iriepa & Navizet, 2019; Zhang et al, 2022). The open–closed mechanism posits that the conformation of luciferase affects energy transfer within the active site pocket and, in turn, light color (Nakatsu et al, 2006). The polarity of the active site may also affect color by promoting proton transfer or keto–enol–enolate equilibrium (Ugarova & Brovko, 2002; Ugarova et al, 2005; Hirano et al, 2009; Naumov et al, 2009). Resonance and electron delocalization theory suggest that control over charge delocalization in oxyluciferin dictates the emitted light color (Branchini et al, 2004a). Enol–keto tautomerism of oxyluciferin in response to pH changes has also been implicated as a mechanism of color variations (White et al, 1971, 1980). Another proposed mechanism suggests that color emission variations are due to the intrinsic properties of specific forms of oxyluciferin that are unaffected by intermolecular interactions or polarity (Ando et al, 2008; Ando & Akiyama, 2010; Hiyama et al, 2013; Wang et al, 2013, 2014; Mochizuki et al, 2014; Carrasco-López et al, 2021; Al-Handawi et al, 2022). These hypotheses are not mutually exclusive and could collectively contribute to understanding the color-tuning mechanisms of luciferase. Therefore, a holistic approach integrating chemical, spectroscopic, and biochemical data with luciferase structures is needed to unravel the complexities of bioluminescence color variations.

**Figure S1.**
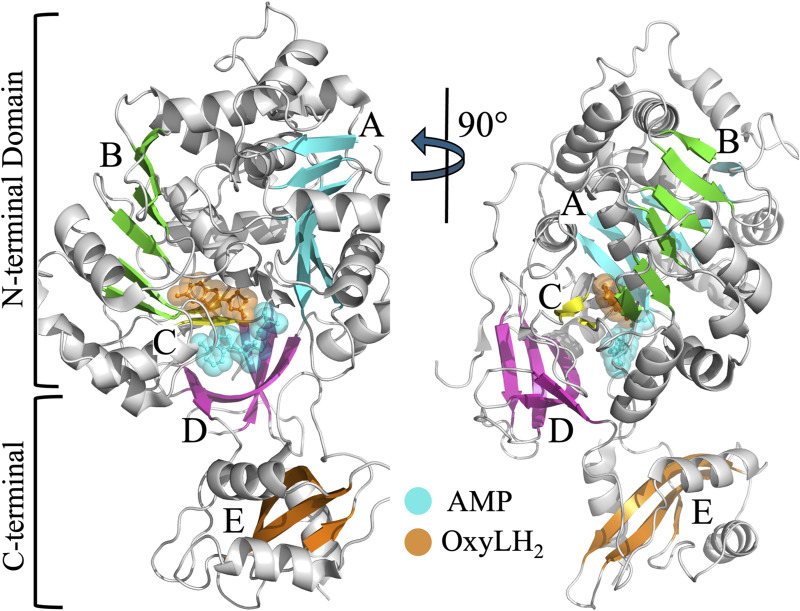
Crystal structure of GB_Av_ (PDB: 6AAA) (1). The cartoon representation depicts the monomeric structure of firefly luciferase, which comprises two main domains: a large N-terminal domain (residues 4–436) and a smaller C-terminal domain (residues 440–544). The two domains are connected by a flexible hinge region (residues 436–440). The N-terminal domain includes three subdomains: β-sheet subdomains-A (cyan) and -B (green), each of which forms a six-stranded β-sheet arranged in a five-layered αβαβα architecture, and a distorted β-barrel subdomain-D (magenta) composed of two three-stranded antiparallel β-sheets. The active site lies within the cleft between the N- and C-terminal domains and is occupied by AMP (cyan) and oxyluciferin (OxyLH_2_, orange), shown in both ball-and-stick and space-filling representations. The ligands were modeled based on their positions in the G_Lc_ structure (PDB: 2D1R). The two-stranded antiparallel β-sheet subdomain-C (yellow) is located between the OxyLH_2_ binding site and subdomain-D. Subdomain-C and the final two strands of the β-sheet subdomains-A and -B form the structural core of the active site. The C-terminal domain contains the three-stranded β-sheet subdomain–E (orange), which acts as a “lid” over the β-barrel of the N-terminal domain, contributing to the closure of the active site. The 90° rotated view illustrates the spatial arrangement of the subdomains and ligand binding within the active site. The structural model was prepared using PyMOL Molecular Graphics System version 2.5.5 (Schrödinger LLC).

Crystal structures of beetle luciferases from different species include the North American *Photinus pyralis* (G_Pp_), the Japanese *Luciola cruciata* (G_Lc_) (both yellow–green light, λmax ≈ 560 nm), *Amydetes vivianii* (GB_Av_) (blue-shifted green light, λ_max_ = 538 nm), and *Phrixothrix hirtus* (RE_Ph_) (orange and red light, λ_max_ = 590–623 nm). All have monomeric structures except RE_Ph_, which is an oligomer of eight subunits (Conti et al, 1996; Franks et al, 1998, Kheirabadi et al, 2013; Nakatsu et al, 2006; Auld et al, 2010; Cruz et al, 2011; Sundlov et al, 2012; Branchini et al, 2017). The monomer structure consists of two distinct domains separated by a wide cleft that houses the active site: a large N-terminal domain and a small C-terminal domain (Fig S1). The larger N-terminal domain (residues 4–436) consists of three subdomains: two β-sheet subdomains and a β-barrel subdomain. The two β-sheets that form β-sheet subdomain-A and subdomain-B are flanked by α-helices and form a five-layered αβαβα tertiary structure (Fig S1). The β-barrel subdomain is distorted, with three sides, including two sides consisting of three-stranded antiparallel β-sheets. The smaller C-terminal domain (residues 440–544) consists of a three-stranded β-sheet and several α-helices. It forms a “lid” over the β-barrel of the N-terminal domain. A flexible hinge region (residues 436–440) connects the two domains and is disordered in many crystal structures. This flexibility allows the closure of the active site in the cleft separating the two domains, thus suggesting a mechanism where substrate binding triggers a conformational change that potentially regulates access to the active site (Nakatsu et al, 2006; Carrasco-López et al, 2018). Interestingly, the apo structure of GB_Av_, which emits blue-shifted green light with λ_max_ = 538 nm, crystallized in space group P2_1_2_1_2_1,_ with two monomers per asymmetric unit. The C-terminal domains of the two monomers display a high degree of flexibility, suggesting different conformations of open and partially closed states (Conti et al, 1996; Nakatsu et al, 2006; Sundlov et al, 2012; Wu et al, 2017; Carrasco-López et al, 2018).

The oligomeric structure of RE_Ph_ is unique among structurally characterized beetle luciferases (Carrasco-López et al, 2018). Two crystals of RE_Ph_ in space groups P1 and P3_1_21 have been analyzed at resolutions of 3.05 and 3.60 Å, respectively (Carrasco-López et al, 2018). In contrast to the monomeric luciferase structures (Conti et al, 1996; Franks et al, 1998; Nakatsu et al, 2006; Auld et al, 2010; Cruz et al, 2011; Sundlov et al, 2012; Kheirabadi et al, 2013; Branchini et al, 2017; Carrasco-López et al, 2018), the P1 and P3_1_21 crystal forms of RE_Ph_ are a tetramer and octamer, respectively (Carrasco-López et al, 2018). In the octameric crystal structure, the N-terminal domains are organized as a tetramer of dimers, with the C-terminal domains extending outward, resulting in structural flexibility and notable thermal motion in these regions. This flexibility makes it difficult to model the C-terminal domains of RE_Ph_ and other beetle luciferases (Auld et al, 2010; Cruz et al, 2011; Thorne et al, 2012; Kheirabadi et al, 2013). For RE_Ph_, only one of the four C-terminal domains in the tetramer and none in the octamer were modeled. However, the octamer structure does provide insights into the dimer and tetramer interfaces of RE_Ph_ (Carrasco-López et al, 2018), which are characterized by different interaction strengths that stabilize the quaternary structure. Multiple hydrogen bonds (H-bonds) stabilize the dimer interface, including side chain interactions of R11 from one monomer with Y26, Y30, and N179 of the other monomer across the dimer interface. These interactions create a robust connection between the two monomers, stabilizing the dimer. By contrast, the tetramer interface, where dimers interact to form tetramers, is stabilized primarily by weaker hydrophobic interactions between key residues from the two dimers, namely, M152, Y153, and F162.

Mutational analyses of beetle luciferases have identified multiple amino acids that can shift the emission color by more than 10 nm, primarily towards the red region of the visible light spectrum. However, many of these mutations alter luciferase expression, activity, or stability or cause non-unimodal emissions (Carrasco-López et al, 2018). Moreover, it has been challenging to identify mutations of RE_Ph_ that induce significant blue shifts of its red-emitting system (Viviani et al, 2008; Carrasco-López et al, 2018). Although residues near the active site, in the C-terminal domain, or at the interface between the two domains are expected to influence the active site microenvironment of RE_Ph_ (Sundlov et al, 2012; Modestova & Ugarova, 2016; Carrasco-López et al, 2018), mutations that blue-shift the color emission often lie far from the active site, preventing a clear mechanistic understanding of their contribution to the color emission of beetle luciferases. Addressing this challenge requires experimental approaches, such as hydrogen/deuterium exchange mass spectrometry (HDX-MS) that are capable of capturing the structural and dynamic consequences of protein mutations in solution.

HDX-MS is a powerful technique for probing protein conformational dynamics with peptide-level resolution. In HDX-MS analysis, acid proteases are used to digest the protein of interest into smaller peptide fragments that are analyzed by liquid chromatography-mass spectrometry (LC-MS) to monitor deuterium uptake at the peptide level. Applications of HDX-MS include single-state analysis, where the focus is on understanding the structural dynamics of a protein in solution (Zhang et al, 1996; Marcsisin et al, 2011; Trabjerg et al, 2017), and multi-state analysis, which compares conformational differences between various protein states or variants (Rist et al, 2003; Rand et al, 2008). By comparing the exchange rates of a WT protein and its mutated or modified counterparts, the structural effects of mutations or chemical modifications can be explored (Hamuro et al, 2002; Iacob et al, 2009). Such analyses have provided details of flexibility and motion missed by static techniques such as X-ray crystallography or cryo-EM (Zhang & Smith, 1993; Jensen & Rand, 2016), enabling groundbreaking hypotheses and insights.

In this study, HDX-MS is used to examine the conformational dynamics of WT and R337L GB_Av_ and WT and L334R RE_Ph_, extending previous work that identified R337L as a red-shift-inducing mutation of GB_Av_ and L334R as a blue-shift-inducing mutation of RE_Ph_. We examine both the apo and ligand-bound forms of these proteins to determine how the mutations and oxyluciferin binding influence the dynamic behavior of these bioluminescent enzymes.

## Results and Discussion

Understanding protein dynamics are essential for elucidating the conformational transitions that enzymes undergo during catalysis, including domain motions, loop rearrangements, and changes in active site accessibility. These structural changes often regulate substrate binding, catalytic efficiency, and product release and play critical roles in enzyme specificity and function. HDX-MS, which leverages liquid chromatography (LC) coupled with mass spectrometry (MS) to monitor the exchange of backbone amide hydrogens with deuterium, is a powerful and versatile tool for probing protein structure, flexibility, and interactions in solution. The rate of deuterium incorporation reflects conformational flexibility, which is modulated by factors such as H-bonding, secondary structure, and local microenvironment. Owing to high sequence redundancy, HDX-MS provides residue-level insights into protein dynamics and solvent accessibility. By comparing exchange rates across different functional states, such as apo, ligand-bound, or mutant forms, HDX-MS can reveal conformational changes linked to enzyme activation, substrate recognition, allosteric regulation, and catalytic turnover. This capability makes HDX-MS an indispensable method for studying the protein motions that govern biological function and mechanistic behavior (Konermann et al, 2011).

In this study, we used HDX-MS to compare the structural dynamics of two WT luciferases: GB_Av_, which emits blue-shifted green light (λ_max_ = 538 nm), and RE_Ph_, which emits red light (λ_max_ = 623 nm). To further elucidate the molecular basis of color tuning, we examined the red-shifted R337L GB_Av_ mutant and the blue-shifted L334R RE_Ph_ mutant. HDX-MS experiments were performed in both the apo and oxyluciferin-bound states to assess conformational changes associated with the binding of substrate (luciferin and ATP). The enzymes were incubated in deuterium oxide (D_2_O) buffer to facilitate hydrogen/deuterium exchange, which was quenched at six time points (0.5, 1.0, 3.0, 6.0 min, 1.0, and 3.0 h) by rapid acidification and cooling, enabling monitoring of exchange at the backbone amides of the peptides. After quenching, the samples were subjected to in-line pepsin digestion and mass spectrometric analysis to quantify deuterium incorporation. For all variants, >99% sequence coverage was achieved, allowing comprehensive mapping of dynamic regions, including those implicated in emission color tuning.

### Identification of peptides underlying color emission and catalysis in GB_Av_ and RE_Ph_ luciferases

**Figure S2.**
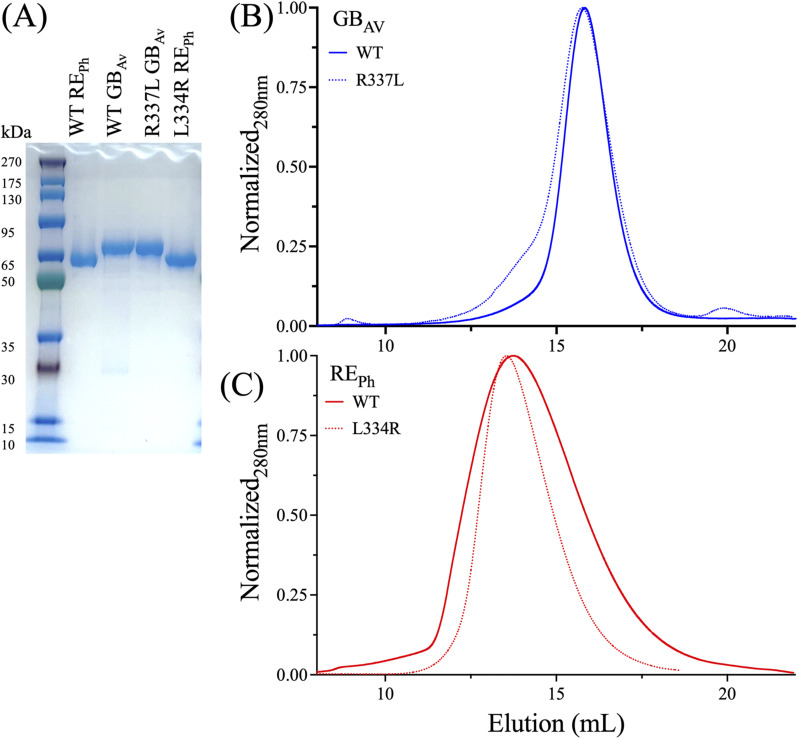
Purification of firefly luciferases. **(A)** SDS–PAGE analysis of purified recombinant WT and mutant of RE_Ph_ and GB_Av,_ confirming >95% purity. **(B, C)** Analytical size-exclusion chromatography of the WT and mutant of GB_Av_ and RE_Ph_. The WT and mutant proteins of GB_Av_ eluted as monomers, while the RE_Ph_ proteins displayed elution patterns consistent with higher oligomeric assemblies, in agreement with previous structural and biophysical analyses (Carrasco-López et al, 2018).

**Figure S3.**
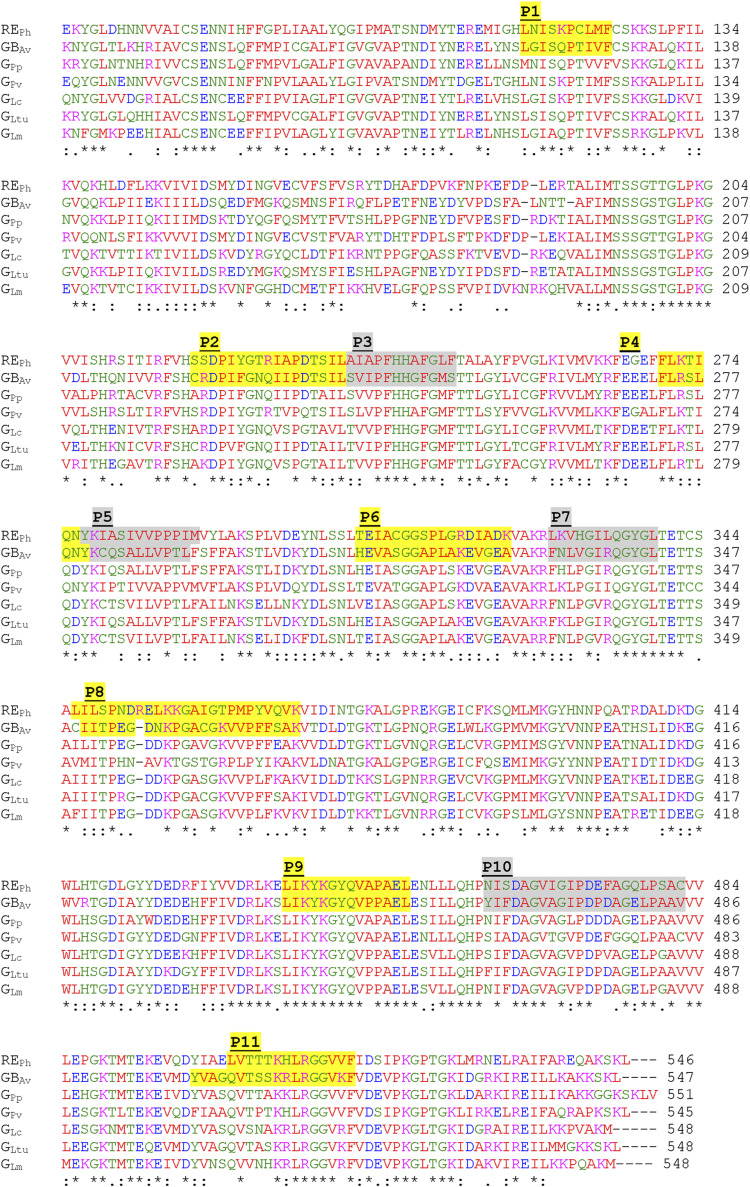
Amino acid sequence alignment of firefly luciferases. A multiple amino acid sequence alignment of RE_Ph_ and GB_Av_ with representative firefly luciferases from various species, including the green-emitting luciferases from the North American firefly *Photinus pyralis* (G_Pp_), *Phrixothrix vivianii* (G_Pv_), Japanese firefly *Luciola cruciata* (G_Lc_), Iranian firefly *Lampyris turkestanicus* (G_Ltu_), and East European firefly *Luciola mingrelica* (G_Lm_), was constructed. Regions corresponding to the 11 peptides identified by HDX-MS that showed significant differences in backbone dynamics between RE_Ph_ and GB_Av_ are highlighted. The highlighted sequences indicate the GB_Av_ peptide regions and their corresponding residue numbers. The sequence alignment was performed using Clustal Omega (EMBL-EBI).

Recombinant expression of both WT and mutant GB_Av_ and RE_Ph_ luciferases was conducted in *Escherichia coli* using N-terminal His_6_-tag as described previously (Carrasco-López et al, 2018). The His-tagged luciferases were further purified using size-exclusion chromatography on a Superdex 200 column (Fig S2B and C), which yielded a final preparation with >95% purity as assessed by SDS–PAGE (Fig S2A). GB_Av_ and RE_Ph_ share ∼50% amino acid sequence identity (Fig S3). Time-resolved HDX-MS revealed distinct deuterium incorporation patterns, with rapid exchange in flexible loops and solvent-exposed regions and slower exchange in structured, solvent-shielded domains. HDX was performed in both apo (unbound) and ligand-bound states (with luciferin and ATP) to probe functional conformational changes associated with substrate binding and catalysis, enabling comparative analysis of structural dynamics. This approach enabled the characterization of luciferases with distinct emission spectra and their color-shifting mutants. Statistical analysis confirmed high reproducibility across replicates, supporting robust time-resolved tracking of the exchange reaction.

Pepsin digestion yielded over 300 overlapping peptides per enzyme, which ranged in length from 7 to 24 residues and collectively covered nearly the entire ∼550 residue sequence. Peptide coverage was extensive, with 329 peptides identified for GB_Av_ and 374 for RE_Ph_. Despite differences in sequence alignment and residue numbering, the peptides mapped to structurally equivalent regions across both enzymes. Whereas all peptides were processed and analyzed during HDX-MS acquisition, only those peptides that exhibited statistically significant differences in deuterium uptake between the red- and green-emitting enzymes, or between apo- and ligand-bound states, were selected for detailed interpretation and visualization in the manuscript.

**Figure S4.**
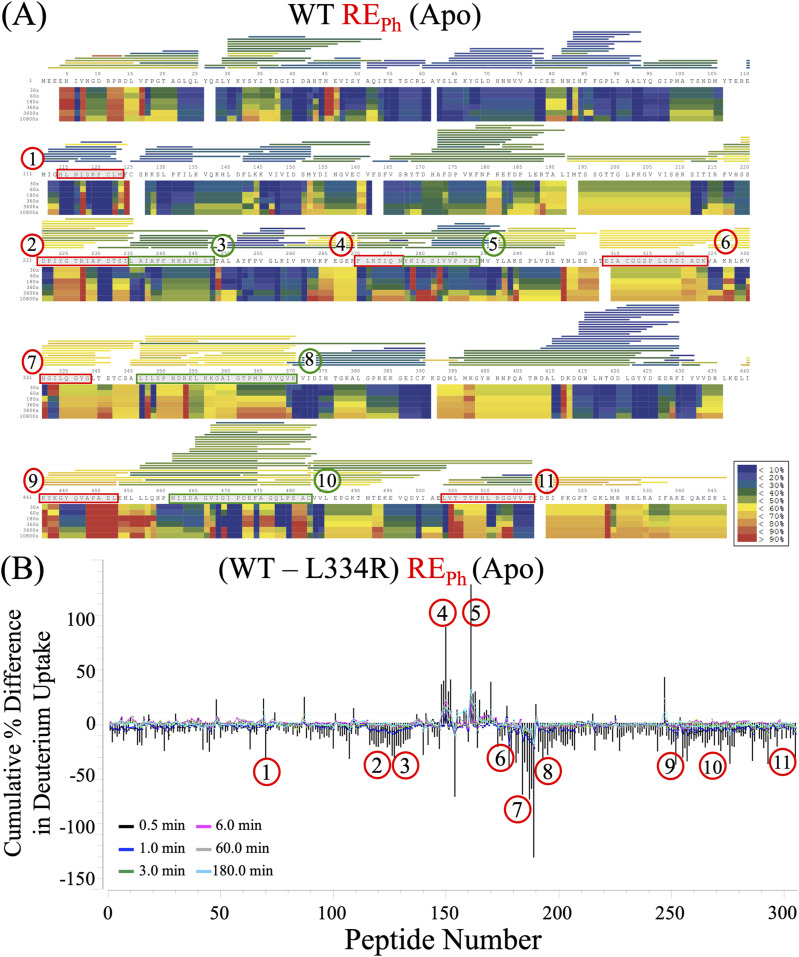
HDX-MS analysis of the apo states of WT and L334R RE_Ph_. **(A)** The heat map shows the time-resolved deuterium uptake profile of WT RE_Ph_ in the apo state across six time points: 0.5, 1.0, 3.0, 6.0 min, 1.0, and 3.0 h. The color scale (blue to red) indicates the relative amount of deuterium uptake at each time point. Amino acid sequences corresponding to regions with the most significant differences in deuterium incorporation are highlighted in green or red. The horizontal bars at the top of the heat map represent the average deuterium incorporation of each peptide identified by pepsin digestion with >90% sequence coverage. The bars are shaded in red/yellow to indicate high deuterium uptake, indicating increased solvent exposure or conformational flexibility. By contrast, blue bars denote limited exchange due to structural protection, such as residues that are buried or engaged in stable hydrogen bonding interactions. **(B)** The residual plot shows the cumulative differences in deuterium uptake between WT and L334R RE_Ph_ in the apo state measured across the six time points. The y-axis represents the cumulative percent difference in deuterium uptake by the peptides. The numbered regions highlight peptides exhibiting the most significant differences in exchange behavior between the WT and mutant enzymes.

**Figure S5.**
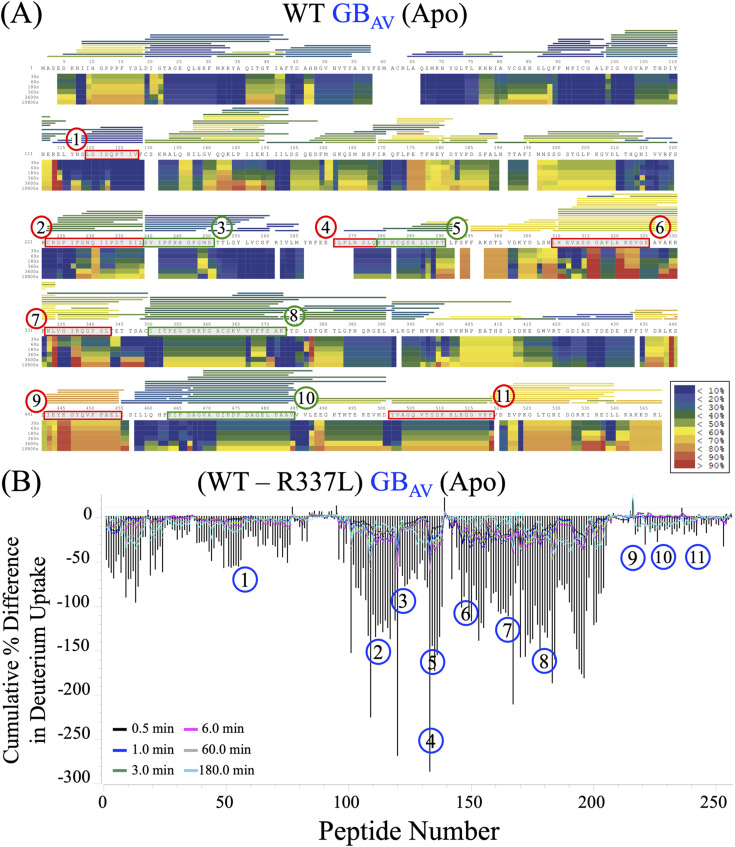
HDX-MS analysis of the apo states of WT and R337L GB_Av_. **(A)** The heat map shows the time-resolved deuterium uptake profile of WT GB_Av_ in the apo state across all measured time points. The color coding and formatting follow that of Fig S3. **(B)** The residual plot shows the cumulative differences in deuterium uptake between WT and R337L GB_Av_ in the apo state measured across the six time points. The y-axis represents the cumulative percent difference in deuterium uptake by the peptides. The numbered regions highlight peptides exhibiting the most significant differences in exchange behavior between the WT and mutant enzymes.

HDX-MS tracks backbone amide deuterium exchange rates, which reflect protection from H-bonding, secondary structure, and conformational fluctuations. Reduced deuterium uptake indicates stronger protection (tighter H-bonding/less opening), although increased uptake indicates weaker protection (greater opening/exposure). Mapping deuterium uptake onto the 3D structures of GB_Av_ and RE_Ph_ revealed domain-specific differences in flexibility and ligand-induced stabilization, with deuterium uptake differences between the two enzymes highlighting regions critical for bioluminescence color tuning. Eleven structurally conserved regions exhibited significant differences in backbone dynamics across eight experimental conditions, including WT and mutant enzymes in both the apo and ligand-bound states. Eight of these regions localized to the N-terminal domain (residues 1–435), in proximity to the active-site cleft, although the other three regions resided in the C-terminal domain (Figs S4A and S5A). Most of the remaining peptides—although successfully identified and quantified—did not exhibit meaningful or reproducible differences between conditions and were therefore not the focus of further structural analysis. The remaining regions displayed minimal differences in deuterium uptake across the GB_Av_ and RE_Ph_ variants. These visualizations underscored the dynamic contrast between the blue-shifted, green-emitting GB_Av_, and the red-emitting RE_Ph_.

Dynamics and deuterium incorporation were greater in the apo states of GB_Av_ and RE_Ph_ than in the ligand-bound states, with the heat maps highlighting areas of high deuterium uptake (red), consistent with solvent-exposed or flexible regions, and low uptake (blue), indicative of structural protection (Figs S4B and S5B). Together, these data provide a high-resolution map of conformational dynamics in beetle luciferases and offer insights into the mechanisms by which ligand binding and sequence variation govern color emission through structural reorganization. For each of the 11 regions that exhibited significant differences in deuterium uptake across the experimental conditions, one representative peptide with the most reliable statistics across five to eight replicates was selected for more detailed analysis.

**Figure S6.**
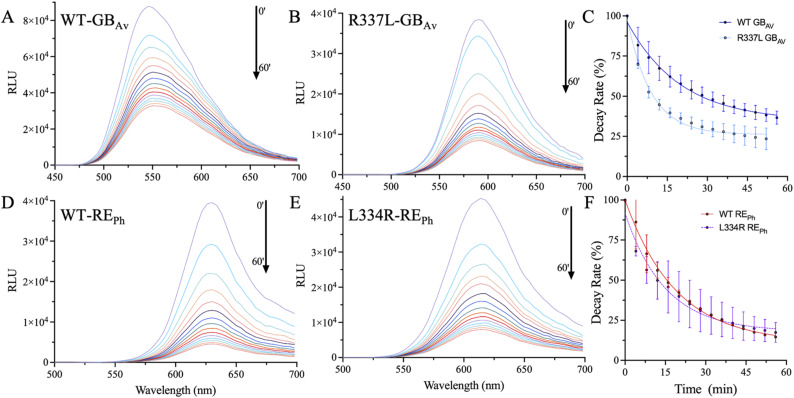
Emission kinetics of WT and mutant RE_Ph_ and GB_Av_. **(A, B, D, E)** Emission scans of GBAv and REPh at 4 min intervals, showing the emission decay for the WT and mutant enzymes. These scans demonstrate the progression of emission intensities under saturated substrate concentrations of 2 mM and 2 μM firefly luciferase enzyme. **(C, F)** Time-decay profiles of the emission intensity at the perspective *λ*_max_ for RE_Ph_ and GB_Av_.

To complement the HDX-MS time courses, we measured emission decay kinetics of the WT and mutant GB_Av_ and RE_Ph_ under saturating substrate concentrations. As expected, kinetic traces show a characteristic decrease in luminescence intensity over time, with distinct *λ*_max_ values for the WT and mutant enzymes that confirm their expected emission colors (Fig S6A–F) (Carrasco-López et al, 2018). Importantly, HDX-MS time courses and emission assays report distinct processes. Whereas HDX-MS probes the rate of deuterium incorporation, reflecting conformational flexibility and solvent accessibility, the emission decay scans reflect the catalytic turnover of the light-producing reaction. The bioluminescence activity observed for all variants confirms the functional integrity of our enzyme preparations used in the HDX-MS experiments.

#### Peptide-1: a distal but dynamically responsive region linking β-sheet–B flexibility to emission color

**Figure S7.**
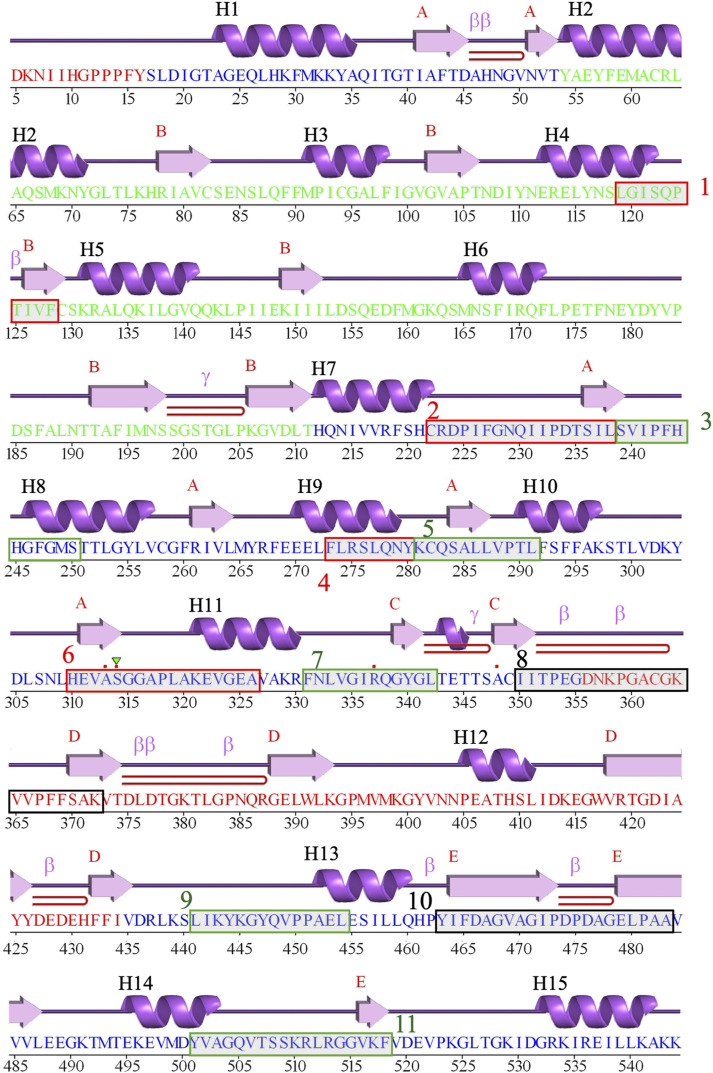
Schematic “wiring diagram” of GB_Av_. The amino acid sequence of GB_Av_ (PDB code: 6AAA) is aligned with the secondary structure elements generated by the PDBsum server (http://www.ebi.ac.uk/thornton-srv/databases/pdbsum/). Secondary structure elements are depicted above the sequence: α-helices are labeled H1-H17, and β-strands are organized according to their respective β-sheet subdomains (A–E). β-turns are also indicated along the sequence. The green–gray boxes highlight regions corresponding to the 11 peptides identified by HDX-MS as contributing to the color emission properties of GB_Av_.

**Figure S8.**
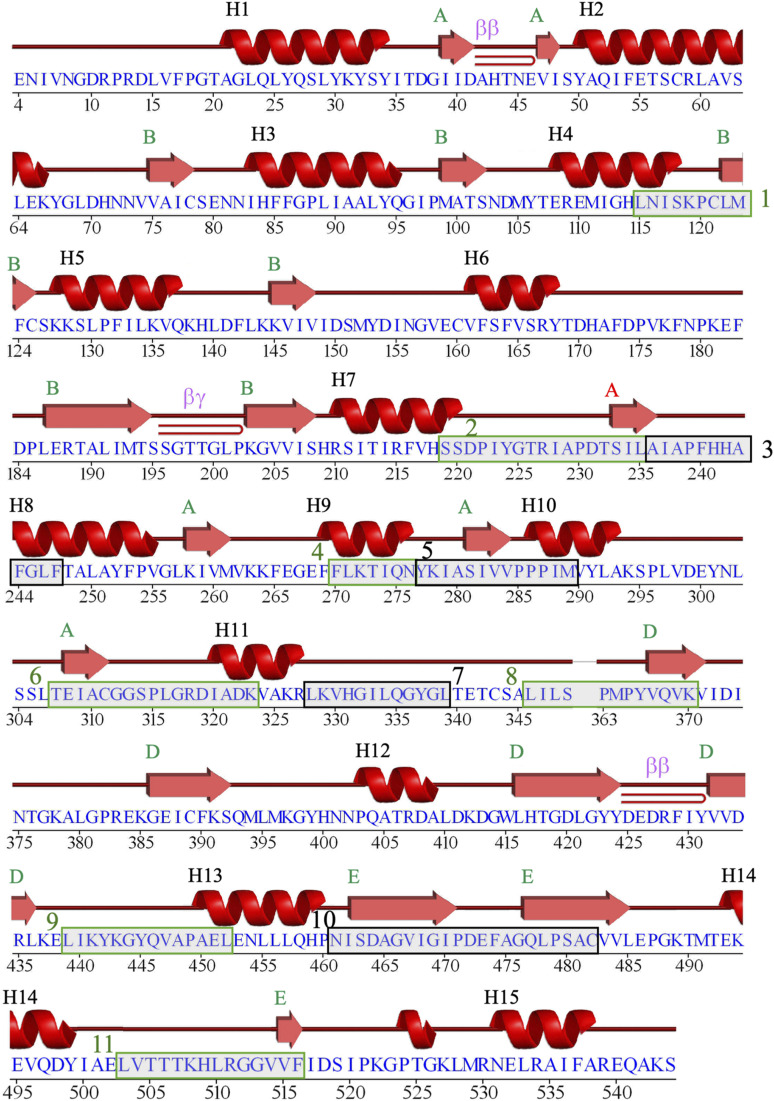
Schematic “wiring diagram” of RE_Ph_. The amino acid sequence of RE_Ph_ (PDB code 6AC3) is aligned with the secondary structure elements generated by the PDBsum server. Secondary structures are represented above the sequence: α-helices are labeled H1-H16, and β-strands are organized according to their respective β-sheet subdomains (A–E). β-turns are also indicated along the sequence. Residues 350–362 are not observed in the electron density map and were not modeled in the crystal structure. Consequently, β-sheet subdomain–C, which is absent from the crystal structure of RE_Ph_, is not represented in the secondary structure elements. To maintain consistent secondary structure labeling between RE_Ph_ and GB_Av_, β-sheet–C has been excluded from the figure numbering. The green–gray boxes highlight regions corresponding to the 11 peptides identified by HDX-MS as contributing to the color emission properties of RE_Ph_.

**Figure 1.**
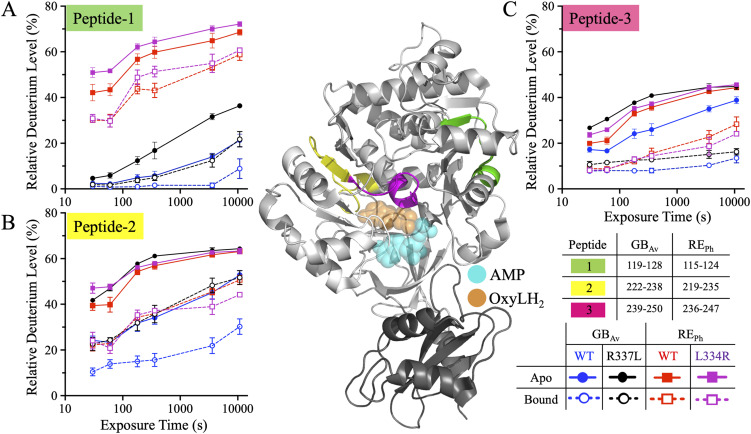
HDX-MS profiles of peptides-1–3 of RE_Ph_ and GB_Av_. The cartoon representation depicts the crystal structure of monomeric GB_Av_ (PDB: 6AAA), with the large N-terminal and small C-terminal domains shown in white and gray, respectively. AMP (cyan) and oxyluciferin (OxyLH_2_, gold) are shown in the active site cavity in ball-and-stick and space-filling formats. The ligands were docked from the G_Lc_ structure (PDB: 2D1R) (Nakatsu et al, 2006). Peptides-1–3 are colored green, yellow, and pink, respectively. The spatial regions of these peptides are identical in both RE_Ph_ and GB_Av_; therefore, only the GB_Av_ cartoon structure is displayed for clarity. HDX-MS was performed at 25°C following incubation of luciferase proteins in D_2_O in both apo (unbound) and ligand-bound (with luciferin and ATP) states. Deuterium incorporation was measured over time for WT and color-shifted mutants of GB_Av_ and RE_Ph_. Solid (—) lines with filled symbols and dashed (– –) lines with hollow symbols indicate the apo and bound states, respectively. Blue and black traces correspond to WT and R337L GB_Av_; red and purple traces correspond to WT and L334R RE_Ph_. Lines are included as guides. **(A)** Peptide-1 is located in the β-sheet–B subdomain and comprises the last turn of the three-turn α-helix–H4, a 12-residue loop, and the third strand of the six-stranded parallel β-sheet–B. It is positioned farthest from the active site. The apo states of WT and L334R RE_Ph_ showed the highest deuterium uptake, which decreased upon ligand binding and in GB_Av_ variants. **(B)** Peptide-2 is part of the β-sheet–A subdomain. It spans the last turn of α-helix H7, a 12-residue loop, and the third β-strand of β-sheet–A. The apo states of WT and L334R RE_Ph_ and R337L GB_Av_ exhibit elevated deuterium uptake, whereas WT GB_Av_ had the lowest incorporation in both states. **(C)** Peptide-3, a structural continuation of peptide-2, comprises a six-residue loop and the first turn of α-helix H8. R337L GB_Av_ had the highest deuterium uptake in the apo state, while WT GB_Av_ had the lowest. In the bound state, WT and L334R REPh had higher uptake than the GB_Av_ variants. The data are the mean ± S.D. of five to eight replicates. The structural figure was generated using PyMOL Molecular Graphics System version 2.5.5 (Schrödinger LLC).

**Figure 2.**
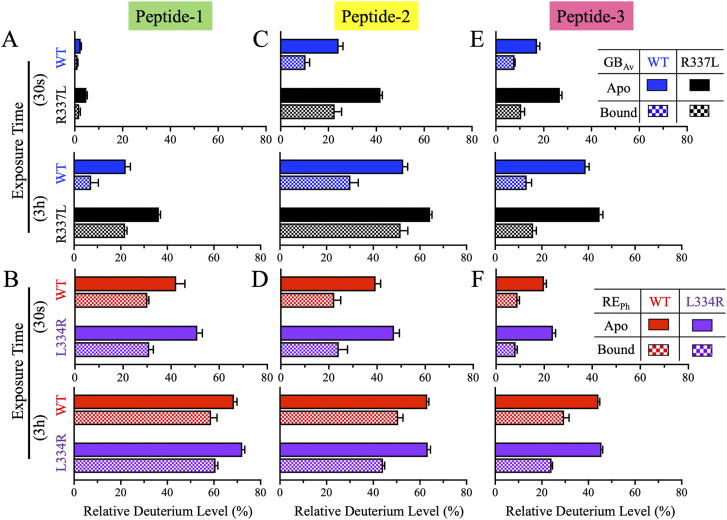
Bar plots of deuterium uptake by peptides-1–3 of RE_Ph_ and GB_Av_ at time points of 0.5 min and 3.0 h. Data for WT and R337L GB_Av_ are colored blue and black, while data for WT and L334R RE_Ph_ are colored red and purple, with data for the apo and bound states indicated by solid and checkered bars, respectively. **(A, C, E)** Relative deuterium uptake by peptides-1, -2, and -3 of GB_Av_ at incubation time points of 0.5 min and 3.0 h. **(B, D, F)** Relative deuterium uptake by peptides-1, -2, and -3 of RE_Ph_ at incubation time points of 0.5 min and 3.0 h. The data are the mean ± S.D. of five to eight replicates.

Peptide-1 (residues 115–124 in RE_Ph_ and 119–128 in GB_Av_) is part of the β-sheet–B subdomain and includes the last turn of α-helix–H4, a 12-residue loop, and the third strand of the six-stranded parallel β-sheet–B (Figs S7 and S8). This is the only dynamic peptide belonging to the β-sheet–B subdomain and is one of the farthest from the active site (Figs 1 and S1). In the apo state, peptide-1 displayed markedly higher deuterium uptake in WT RE_Ph_ than in WT GB_Av_, with average differences of ∼45% (Figs 1A and 2A and B), indicating greater flexibility in the red-emitting RE_Ph_ than in the green-emitting GB_Av_. Comparison of R337L GB_Av_, which red-shifts emission by 42 nm (from 538 to 580 nm), with WT GB_Av_ further supports this correlation between red emission and enhanced dynamics. Whereas deuterium uptake by peptide-1 at 0.5 min was similar between WT and R337L GB_Av_, the R337L mutant showed ∼15% higher deuteration at 3.0 h in both the apo and bound states (Figs 1A and 2A), indicating a delayed but significant increase in flexibility. Conversely, L334R RE_Ph_, which blue-shifts emission by 18 nm (from 623 to 605 nm), showed only minor increases in deuterium uptake by peptide-1 of 9% at 0.5 min and 3% at 3.0 h relative to WT RE_Ph_ in the apo state (Fig 2A and B), and no significant differences were observed in the bound state.

In the presence of substrate, deuterium uptake by peptide-1 decreased in all enzymes. For WT GB_Av_, the largest drop in uptake, from 21% to 9%, was observed at 3.0 h (Figs 1A and 2A), whereas WT RE_Ph_ showed a consistent decrease of 10% at all time points (Figs 1A and 2B). Despite these reductions, WT RE_Ph_ acquired 30% and 50% higher deuterium uptake compared with WT GB_Av_ in the bound state at 0.5 min and 3.0 h, respectively, indicating that RE_Ph_ retained significantly greater dynamics even in the bound state. Collectively, these results indicate that peptide-1, though located distantly from the active site, exhibits dynamic behavior that correlates with the emission color and structural flexibility of luciferase. The increased flexibility observed in red-emitting enzymes, especially in R337L GB_Av_ and WT RE_Ph_, suggests that distal regions like peptide-1 may contribute to global conformational dynamics that influence bioluminescence properties.

Peptide-1 is unique among the dynamic regions highlighted in this study in that it is spatially distant from the catalytic core yet exhibits distinct and reproducible dynamics. Despite its separation from the active site, Peptide-1 consistently displayed elevated deuterium uptake in both WT and L334R REPh variants across apo and ligand-bound states, with low experimental error across replicates. These dynamics were not observed in any other distal peptides, none of which showed statistically significant or reproducible differences between the red- and green-emitting enzymes. Its dynamic behavior, despite being distant from the active site, suggests a potential allosteric link to emission color and may contribute to global conformational changes affecting bioluminescence.

#### Peptides-2 and -3: a conserved, dynamically tuned active site region governing luciferin binding and emission color

Peptides-2 and -3 are contiguous regions within the β-sheet–A subdomain. Peptide-2 (residues 219–235 in RE_Ph_ and 222–238 in GB_Av_) comprises the final turn of α-helix-H7, a 12-residue loop, and the third β-strand of the six-stranded β-sheet-A, whereas peptide-3 (residues 236–247 in RE_Ph_ and 239–250 in GB_Av_) continues with a six-residue loop and the first turn of α-helix-H8 (Figs 1, S1, S7, and S8). Together, these peptides shape one side of the active site cleft and are structurally conserved among beetle luciferases. In peptide-3, residues H242, F244, and T248 in RE_Ph_ (H245, F247, and T251 in GB_Av_) are highly conserved among beetle luciferases (Fig S3) and are positioned within 4 Å of the luciferin and AMP binding pockets, where they participate in key interactions that stabilize substrate binding. Specifically, the imidazole of H242 forms a 2.7 Å H-bond with the α-phosphate of AMP, whereas F244 and T248 make van der Waals contacts with the benzothiazole ring of luciferin from opposite sides. An internal H-bond between T248 and the backbone of F244 (2.7 Å) further reinforces this interaction network.

In the apo state, deuterium uptake by both peptides at early time points was consistently higher for WT RE_Ph_ than WT GB_Av_. For peptide-2, uptake at 0.5 min was 40% for WT RE_Ph_ versus 25% for WT GB_Av_ (Figs 1B and 2C and D), whereas peptide-3 deuteration was similar (∼20%) for WT GB_Av_ and WT RE_Ph_. Interestingly, uptake by peptide-2 in R337L GB_Av_, which has red-shifted emission, was similar to that in WT RE_Ph_, suggesting that increased flexibility around the active site is linked to red emission (Figs 1B and 2D). For peptide-3, deuterium uptake was similar for both WT and L334R RE_Ph_ as well as R337L GB_Av_ (Figs 1C and 2E and F). The apo deuteration profile of WT GB_Av_ resembled the bound profile of WT RE_Ph_, reinforcing the notion that RE_Ph_ has inherently higher dynamics even when substrates are bound. Peptide-3 also showed an apparent time-dependent increase in deuteration, doubling from ∼20% at 0.5 min to ∼40% at 3.0 h in all enzymes.

Upon substrate binding, deuterium uptake by peptides-2 and -3 decreased in all variants, but the same general pattern persisted: WT and L334R RE_Ph_ and R337L GB_Av_ exhibited higher uptake than WT GB_Av_. These trends support the broader model that red-emitting luciferases maintain higher structural dynamics around the active site. Notably, the dynamics of peptides-2 and -3 in L334R RE_Ph_, which has blue-shifted emission, were similar to those of WT RE_Ph_ in the apo and bound states. Together, the results for peptides-2 and -3 underscore the role of β-sheet–A and α-helix-H8 dynamics in modulating luciferin interactions and tuning the emission color of beetle luciferases.

These two peptides form a continuous structural unit within the β-sheet–A subdomain, in close proximity (∼8.0 Å) to the benzothiazole moiety of luciferin. Together, they constitute a key region of the luciferin binding pocket that defines the base of the active site cleft and plays essential roles in substrate orientation and stabilization. Numerous studies have emphasized the functional importance of peptide-2, particularly loop^223–235^ GB_Av_ or loop^220–232^ RE_Ph_, controlling the spectral properties of beetle luciferases (Viviani et al, 2001, 2002, 2008). For example, T226, which is located centrally within this loop, is a critical determinant of emission color across luciferases from diverse species. In GB_Av_, T226N substitution produces a marked red shift in emission from 546 to 574 nm at pH 8.0 (Viviani et al, 2002). A more moderate red shift from 534 to 546 nm is observed in the T226N mutant of *Pyrearinus termitilluminans* luciferase (GL_Pt_) (Viviani et al, 2002). Notably, *Pyrocoelia miyako* luciferase (G_Pm_) exhibits a substantial red shift from 547 to 604 nm upon introduction of T226N (Viviani et al, 2002). By contrast, in RE_Ph_, the T226N substitution resulted in a blue shift in emission from 622 to 611 nm at pH 8.0, highlighting the species-specific role of T226 in modulating bioluminescence emission (Viviani et al, 2002). Similarly, T226F substitutions resulted in red-shift emissions as far as 590 nm in *Ragophthalmus ohbai* luciferase (G_Ro_) and more modestly from 534 to 546 nm in GL_Pt_ (Viviani et al, 2002, 2008). These observations underscore the role of T226 in loop stabilization and emission tuning, which likely includes modulating interactions with luciferin and the surrounding solvent-exposed channel. Y227 has also been shown to impact emission properties. In *Macrolampis sp2* luciferase (GL_Ms_), the Y227F mutation alters the native yellow emission (569 nm) to a bimodal spectrum with peaks at 569 and 618 nm (Viviani et al, 2001), reflecting a red shift and suggesting a role in fine-tuning the polarity or steric properties of the binding pocket.

Peptide-3 continues this dynamic region and includes the highly conserved H245 (H242 in RE_Ph_), a residue whose pivotal role in emission tuning has been extensively characterized. Located in α-helix-H8, H245 lies at the edge of the luciferin binding pocket near the carboxylate group of luciferin and the γ-phosphate of ATP. In *P. pyralis* luciferase (G_Pp_), H245A leads to a substantial red shift from 557 to 604 nm, and similar shifts are induced by other substitutions (e.g., H245D: 617 nm; H245N: 613 nm). These effects imply roles of H245 in stabilizing specific oxyluciferin conformers and maintaining an optimal electrostatic microenvironment (Branchini et al, 1998, 1999, 2003; Ugarova & Brovko, 2001; Tafreshi et al, 2008). The aromatic residue F247 (RE_Ph_: F244), located within van der Waals distance of luciferin’s benzothiazole ring, has also been shown to influence emission color. In G_Pp_, the F247A mutation red-shifts emission from 557 to 587 nm, whereas the F247Y mutant preserves WT-like color and enzymatic function, indicating that π-stacking and aromatic character at this position are important for efficient catalysis and color specificity (Ugarova & Brovko, 2001; Branchini et al, 2003; Tafreshi et al, 2008).

Beyond individual mutations, combinatorial mutagenesis studies have confirmed the cooperative role of residues in peptide-3. The S246H/H347A double mutant of GL_Pt_ red-shifts emission from 538 to 602 nm (Nishiguchi et al, 2015), whereas a triple mutant (R214K/S246H/H347A) increases this shift to 619 nm. The quadruple mutant (R214K/H241K/S246H/H347A) increases red-shifts emission by up to 88 nm to reach 626 nm (Nishiguchi et al, 2015). These cumulative effects demonstrate how multiple local interactions within this peptide region can synergistically reshape the emission landscape. The effects of the nearby residue Y255, although not part of peptide-3, further support the broader importance of this region. Y255 is located ∼7.0 Å from luciferin and influences the H-bonding network at the base of the pocket. In G_Pp_, the Y255F mutation induces a 13-nm red shift (560–573 nm), while the reverse substitution (F255Y) in *Photinus scintillans* luciferase (P_sn_) produces a 9-nm blue shift (575 to 566 nm) (Branchini et al, 2017), reflecting the role of this residue in modulating active site polarity.

Together, peptides-2 and -3 represent a structurally conserved yet dynamically responsive region of the luciferase scaffold. They harbor multiple residues that play critical roles in stabilizing the substrate, shaping the electrostatic environment of the chromophore, and ultimately dictating emission color. The HDX-MS data and the results of mutational analyses congruently highlight this region as a functional hub for color modulation in beetle luciferases.

#### Coordinated dynamics of peptides-4 and -5 in the β-sheet-A subdomain modulate active site flexibility and emission color

**Figure 3.**
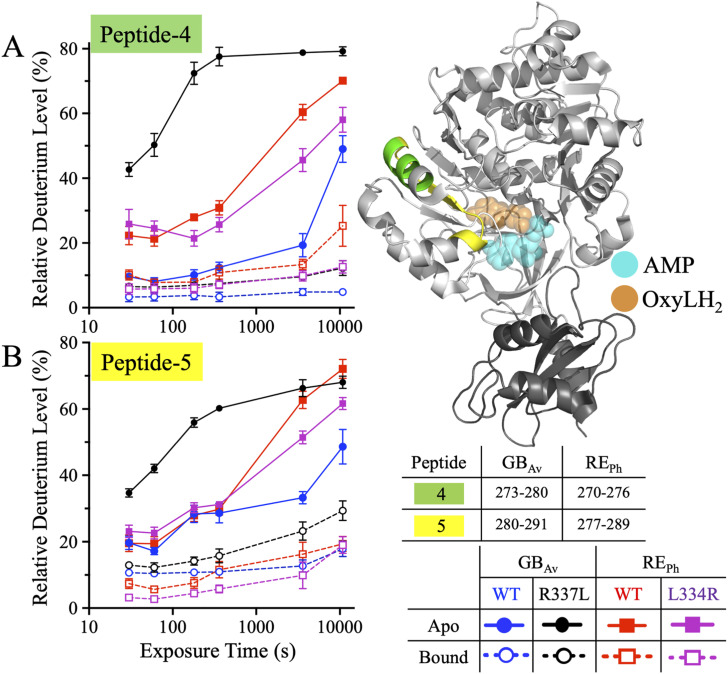
HDX-MS profiles of peptides-4 and -5 of RE_Ph_ and GB_Av_. The cartoon representation depicts the crystal structure of monomeric GB_Av_ (PDB: 6AAA) as described in Fig 1. Peptides-4–6 are colored green, yellow, and pink, respectively. The spatial regions of these peptides are identical in both RE_Ph_ and GB_Av_; therefore, only the GB_Av_ cartoon structure is displayed for clarity. The format of the deuterium uptake graphs follows that of Fig 1. **(A)** Peptide-4 is part of the β-sheet–A subdomain. In the apo state, R337L and WT GB_Av_ had the highest and lowest deuterium uptake. In the bound state, all enzymes had similar deuterium levels, with WT RE_Ph_ acquiring more deuterium than WT GB_Av_. **(B)** Peptide-5, which is a continuation of peptide-4, is part of the β-sheet–A subdomain. In the apo state, the deuterium incorporation of peptide-5 was similar to that of peptide-4. In the bound state, peptide-5 had the highest deuterium incorporation in R337L GB_Av_ and the lowest in L334R RE_Ph_. The data are the mean ± S.D. of five to eight replicates. The structural figure was prepared using PyMOL Molecular Graphics System version 2.5.5 (Schrödinger LLC).

**Figure S9.**
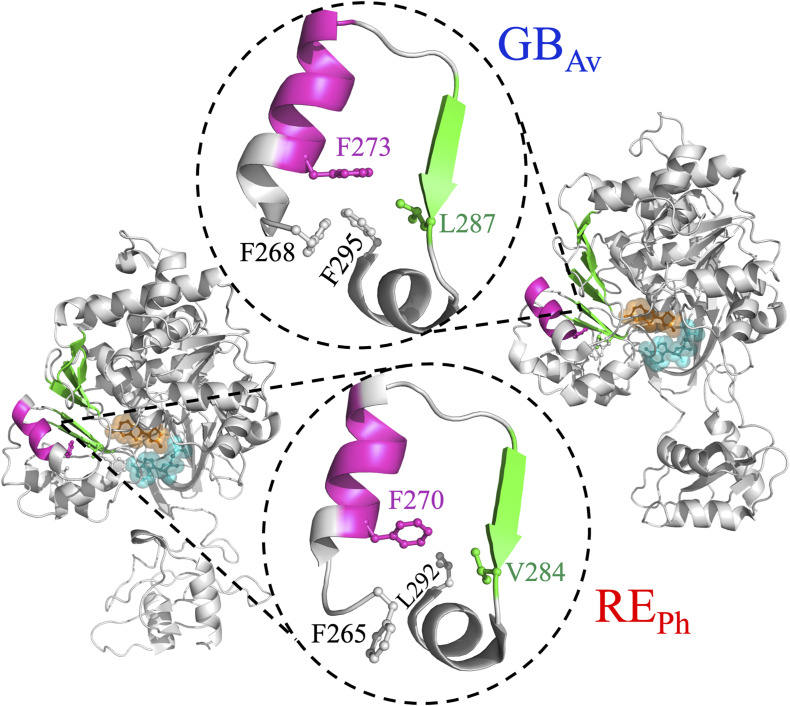
Hydrophobic pocket formed by peptides-4 and -5. The cartoon representation depicts the αβα-motif that forms a hydrophobic pocket in RE_Ph_ and GB_Av_. The active site is occupied by AMP (cyan) and OxyLH_2_ (orange), displayed in both ball-and-stick and space-filling representations. In GB_Av_, a well-defined hydrophobic pocket is formed through tight packing of the side chains of F268 and F273 (α−helix-H9, pink), L287 (the fifth β-strand of β-sheet-A, green), and F295 (α-helix-H10, white). By contrast, the corresponding motif in RE_Ph_ does not form a comparably defined pocket: F265 points away from the hydrophobic core, while F270, V284, and L292 provide limited hydrophobic interactions. The presence of a longer side chain in L287 (GB_Av_) compared with the shorter side chain of V284 (RE_Ph_) could result in stronger van der Waals interactions, thereby enhancing the hydrophobic packing in GB_Av_. Additionally, the outward orientation of F265 in RE_Ph,_ away from the hydrophobic pocket, further reduces its compactness and stability compared with GB_Av_. The figure was prepared using PyMOL Molecular Graphics System version 2.5.5 (Schrödinger, LLC).

Peptides-4 and -5 span sequential regions of the β-sheet-A subdomain and together form a stabilizing surface behind the active site cleft. With a length of seven residues, peptide-4 (residues 270–276 in RE_Ph_ and 273–280 in GB_Av_) is the smallest of the 11 peptides analyzed in detail and makes the last turn of the three-turn α-helix-H9 (Figs 3 and S1). Peptide-5 (residues 277–289 in RE_Ph_ and 280–291 in GB_Av_) is 11 residues long and includes a four-residue loop, the fifth β-strand of β-sheet-A, a three-residue loop, and the first turn of α-helix-H10 (Figs 3, S7, S8, and S9). These structural elements are positioned adjacent to the substrate-binding pocket and contribute to maintaining the geometry of the active site.

**Figure 4.**
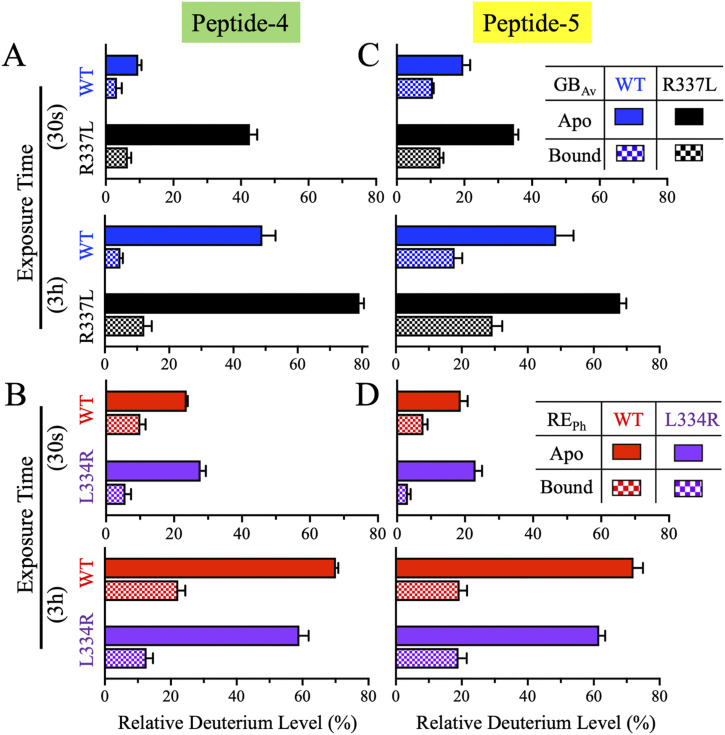
Bar plots of deuterium uptake by peptides-4 and -5 of RE_Ph_ and GB_Av_ at time points of 0.5 min and 3.0 h. The plots are formatted similarly to those in Fig 2. **(A, B)** Relative deuterium uptake by peptides- 4 and -5 of GB_Av_ at incubation time points of 0.5 min and 3.0 h. **(C, D)** Relative deuterium uptake by peptides-4 and -5 of RE_Ph_ at incubation time points of 0.5 min and 3.0 h. The data are the mean ± S.D. of five to eight replicates.

In the apo state, WT and L334r RE_Ph_ exhibited similarly low deuteration of peptide-4 at 0.5 min (∼25%), which remained unchanged until 6 min and gradually increased to 70% and 58%, respectively, by 3 h (Figs 3A and 4C). WT GB_Av_ exhibited lower initial labeling (∼10%) and a slower increase, reaching only 50% at 3 h (Figs 3A and 4A). R337L GB_Av_ showed substantially elevated deuterium uptake by peptide-4 from the outset, with 42% labeling at 0.5 min, which increased rapidly and plateaued near 80% at 6 min, exceeding even WT RE_Ph_, indicating significantly greater flexibility.

The dynamics of peptide-5 were similar to those of peptide-4. In the apo state, WT RE_Ph_ and GB_Av_ exhibited comparable deuteration trends up to 6 min. However, by 1 and 3 h, WT RE_Ph_ reached higher deuterium levels (63% and 72%) than WT GB_Av_ (33% and 49%), consistent with the greater structural dynamics of the red-emitting variant (Figs 3B and 4B and D). R337L GB_Av_ again displayed markedly enhanced exchange, rising from 35% at 0.5 min to 69% at 3 h. The deuteration of peptide-5 in L334R RE_Ph_ was comparable to that in WT RE_Ph_ at up to 6 min but was ∼11% lower at 1 and 3 h, indicating reduced dynamics in this region.

In the substrate-bound state, both peptides exhibited significantly reduced deuterium incorporation across all variants, consistent with structural stabilization upon ligand binding. Peptide-4 deuteration was slightly lower than that of peptide-5, with the highest exchange observed in R337L GB_Av_. Taken together, the HDX-MS profiles of peptides-4 and -5 highlight a coordinated region of dynamic tuning near the active site cleft. The R337L mutation of GB_Av_ significantly enhances flexibility, whereas the L334R mutation of RE_Ph_ leads to a general reduction in dynamics. These differences correlate with the spectral properties of each enzyme and support a model in which red emission is associated with a more dynamic active site environment, whereas blue emission is favored by increased rigidity.

β-sheet-A forms the active site; therefore, its conformation should be critical for the color emission of beetle luciferases. Both peptides-4 and -5 are part of an αβα-motif that includes α-helix-H9, the fifth strand in β-sheet-A, and the first turn of α-helix-H10 (Figs S7, S8, and S9). The αβα-motif facilitates different bonding interactions in RE_Ph_ compared with GB_Av_, which may explain the differences in conformational dynamics observed in the HDX-MS data. However, the van der Waals bonding interactions of the αβα-motif are weaker in RE_Ph_ than in GB_Av_; this discrepancy is attributable to multiple factors. First, the orientations of two phenylalanines that are highly conserved in all beetle luciferases, F265 and F270 in RE_Ph_ and F268 and F273 in GB_Av_, differ (Fig S3). In RE_Ph_, F265 points away from the hydrophobic pocket and does not interact with the side chains of F270, V284, and L292 (Fig S9). By contrast, the αβα-motif of GB_Av_ forms a tight hydrophobic pocket in which the side chain of F268 points toward and forms tight van der Waals bonding interactions with F273 of α-helix-H9, L287 of β-sheet-A, and F295 of α-helix-H10 (Fig S9). Second, the large hydrophobic surface of the benzyl side chain of F295 in GB_Av_ is replaced by the short isobutyl side chain of L292 in RE_Ph_, which is not expected to participate in establishing a strong hydrophobic pocket. Finally, the larger isobutyl side chain of L287 in GB_Av_ is replaced with the smaller isopropyl side chain of V284 in the αβα-motif of RE_Ph_. The latter is not expected to initiate strong van der Waals bonding interactions with the hydrophobic pocket of RE_Ph_.

Studies have shown that this region plays an important role in the color emission of beetle luciferases, and the dynamic data for peptide-5 support the critical role of the S284 position in modulating the emission properties of beetle luciferases. For example, the S284T mutation red-shifts the green emission maximum of WT *Lampyris turkestanicus* luciferase (GL_Tu_) from 555 to 620 nm (Tafreshi et al, 2008). In G_Pp_, introducing the S284T mutation red-shifts the green emission from 557 to 616 nm (Caysa et al, 2009). Additional amino acid substitutions at S284, including S284N, S284H, and S284I, result in red-shifted emission with λ_max_ values of 608, 611, and 616 nm, respectively. Among these, the S284T mutant is the most promising red-emitting variant, as its emission bandwidth remains narrow and its enzymatic efficiency is superior to that of the S284N, S284H, and S284I mutants (Branchini et al, 2005b). The red shift in these mutants is attributed to alterations in the luciferase active site that likely modify the electronic environment of the excited oxyluciferin, stabilizing a lower energy emissive state (Branchini et al, 2005b). Combining the S284T mutation with V241I and I351A mutations further optimizes the red shift and kinetic properties of luciferase. In G_Lc_, this position is S286, and the S286N mutation red-shifts the emission spectrum (λ_max_) from 560 to 605 nm (Kajiyama & Nakano, 1991; Carrasco-López et al, 2018). Highlighting this region’s importance in color emission, the mutations I288A and I288V produce an even greater red shift to 613 nm.

#### Dynamics of active site proximal peptides-6, -7, and -8 in beetle luciferases

Peptides-6, -7, and -8 define a contiguous region that lines the core of the luciferin-binding cavity in beetle luciferases and contributes directly to shaping the bioluminescence emission spectrum. These peptides form an interwoven structural network around the active site cleft that anchors key elements of the β-sheet-A, β-sheet-C, and β-sheet-D subdomains and supports crucial loop regions.

**Figure 5.**
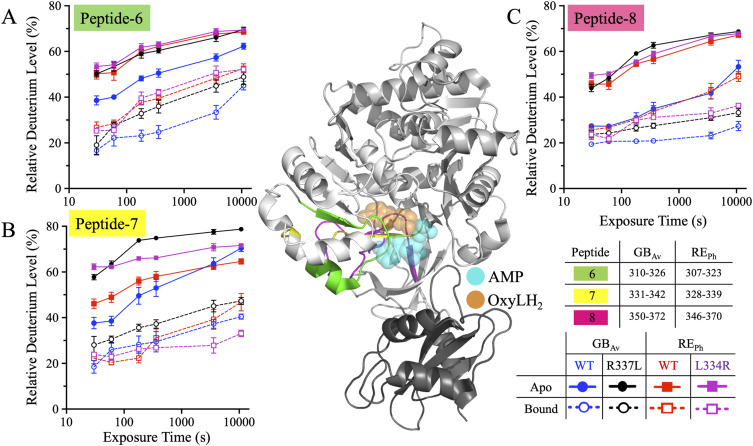
HDX-MS profiles of peptides-6–8 of RE_Ph_ and GB_Av_. The cartoon representation depicts the monomeric GB_Av_ structure (PDB: 6AAA) as described in Fig 1. Peptides-7 and -8 are colored green and yellow, respectively. The spatial regions of these peptides are identical in both RE_Ph_ and GB_Av_; therefore, only the GB_Av_ cartoon structure is displayed for clarity. The format of the deuterium uptake graphs follows that of Fig 1. **(A)** Peptide-6 comprises the last strand of β-sheet-A and the first two turns of α-helix-H11. Deuterium incorporation was highest in the apo state for all enzymes except WT GB_Av_, which had lower dynamics. A similar pattern of deuterium incorporation was observed in the bound state, with WT GB_Av_ displaying the lowest deuterium incorporation. **(B)** Peptide-7 comprises a 12-residue loop and the first strand of the small two-stranded antiparallel β-sheet-C. All variants exhibited higher deuterium uptake in the apo state relative to the bound state, indicating increased flexibility. Among all enzymes, L334R RE_Ph_ and R337L GB_Av_ had the highest deuterium uptake in the apo state. **(C)** Peptide-8, the largest peptide identified in the HDX-MS analysis, includes the second strand of the two-stranded antiparallel β-sheet-C, a 12-residue connecting loop, and the first β-strand of the four-stranded antiparallel β-sheet-D. Deuterium uptake was highest in the apo states of WT and L334R RE_Ph_ and R337L GB_Av_, whereas WT GB_Av_ had the lowest dynamics. Notably, the apo state of WT GB_Av_ had deuterium incorporation levels comparable to those of the bound state of WT RE_Ph_. The structural figure was generated using PyMOL Molecular Graphics System version 2.5.5 (Schrödinger LLC). The data are the mean ± S.D. of five to eight replicates.

**Figure 6.**
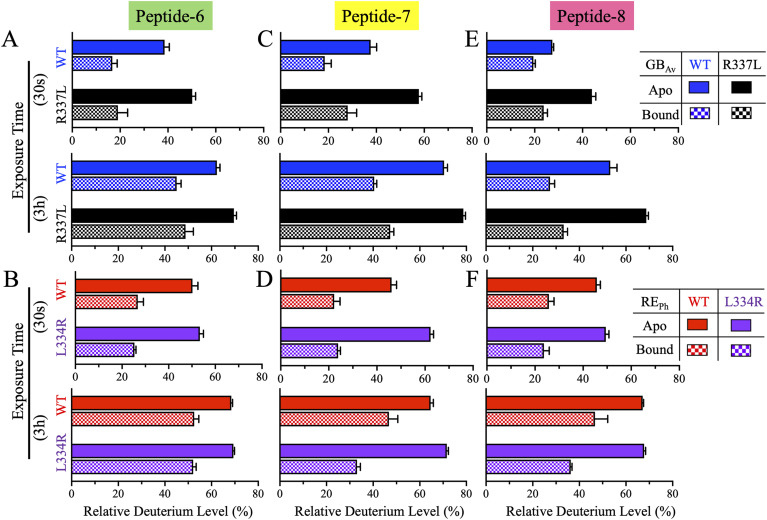
Bar plots of deuterium uptake by peptides-6–8 of RE_Ph_ and GB_Av_ at time points of 0.5 min and 3.0 h. The plots are formatted similarly to those in Fig 2. **(A, C, E)** Relative deuterium uptake by peptides-6–8 of GB_Av_ at incubation time points of 0.5 min and 3.0 h. **(B, D, F)** Relative deuterium uptake by peptides-6–8 of RE_Ph_ at incubation time points of 0.5 min and 3.0 h. The data are presented as the mean ± S.D. of five to eight replicates.

Peptide-6 (residues 307–323 in RE_Ph_ and 310–326 in GB_Av_) comprises the terminal β-strand of β-sheet-A, a five-residue loop, and the initial two turns of the three-turn α-helix-H11. It participates directly in the active site architecture, with the side chain of S314 (GB_Av_) and the corresponding C311 (RE_Ph_) positioned within H-bonding distance (∼3.2 Å) of the 6′-hydroxyl group of the benzothiazole ring of luciferin, suggesting a potential role in shaping the microenvironment of the substrate-binding pocket. The ligands, luciferin and ATP, were docked into the apo structures of RE_Ph_ and GB_Av_ using the *P. pyralis* G_Lc_ complex (PDB: 2D1R) to identify their active sites (Nakatsu et al, 2006). HDX-MS revealed that in the apo state, peptide-6 of WT RE_Ph_ exhibited 50% deuteration at 0.5 min, which gradually increased to 70% by 3 h (Figs 5A and 6B). This contrasts with WT GB_Av_, which showed lower deuterium uptake by peptide-6 (39% → 62%) across the same time points, indicating greater rigidity (Figs 5A and 6A). Ligand binding reduced the dynamics of peptide-6 in both enzymes to different extents: deuteration of peptide-6 decreased to 27–53% in RE_Ph_ and reached only 17–45% in GB_Av_. These results align with the model in which blue-shifted emission correlates with rigid, less dynamic structures, whereas red-shifted emission arises from a more flexible active site environment.

Strikingly, the dynamics of peptide-6 in R337L GB_Av_, in which emission is red-shifted by 42 nm, mirrored that in WT RE_Ph_ (50% → 70% uptake in apo state) (Figs 5A and 6A). By contrast, the dynamics of peptide-6 in L334R RE_Ph_, in which emission is blue-shifted by 18 nm, did not differ significantly from that in WT RE_Ph_ (Figs 5A and 6B). These findings suggest that increasing peptide-6 flexibility contributes to red-shifted emission, whereas reducing dynamics in this region may not be sufficient to induce blue shifts without broader structural consequences. Peptide-6 thus represents a functionally sensitive node at which local flexibility impacts substrate alignment and the electronic environment to influence the energy of light emission.

Peptide-7 (residues 328–339 in RE_Ph_ and 331–342 in GB_Av_) immediately follows peptide-6 and forms a 12-residue loop that links α-helix-H11 to the first strand of antiparallel β-sheet-C (Figs 5, S7, and S8). This loop plays a key role in shaping the active site cleft, contributing to both the luciferin and ATP binding pockets. Five residues that line the catalytic cavity—Q335, G336, Y337, G338, and L339 (RE_Ph_)—are conserved across beetle luciferases (Fig S3). Structural analyses, including that of G_Lc_, have shown that Y337 is oriented away from the luciferin-binding pocket toward the ATP-binding site, with its hydroxyl group ∼4.6 Å from the ribose 3′-OH of AMP, suggesting involvement in the adenylation step (Nakatsu et al, 2006; Carrasco-López et al, 2018). HDX-MS analysis revealed that peptide-7 in both WT luciferases showed similar 10–30% increases in deuteration from 0.5 min to 3 h in both the apo and bound states (Figs 5B and 6C and D). Across all variants, deuterium uptake was 20–40% higher in the apo state, reflecting ligand-induced stabilization. Notably, R337L GB_Av_ exhibited the highest deuterium incorporation in peptide-7 in the apo state, indicating enhanced flexibility in this catalytically critical region. WT GB_Av_ exhibited the lowest uptake in peptide-7 in both states, reflecting a more rigid loop that may support high-energy (blue-shifted) emission. These findings reinforce the trend that red emission is associated with elevated flexibility, particularly in regions near the active site cleft. The lack of a significant reduction in peptide-7 dynamics in L334R RE_Ph_ relative to WT RE_Ph_ again suggests that red-shift reversal may require broader or more cooperative structural stiffening.

**Figure S10.**
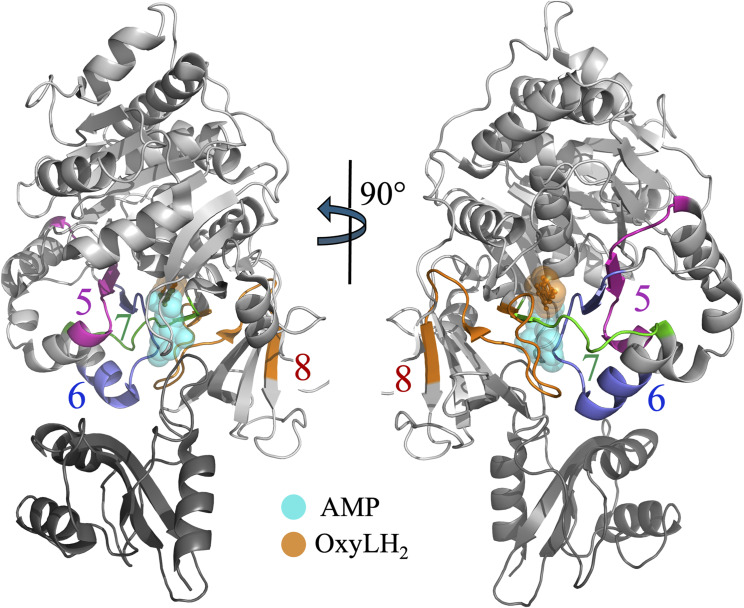
Peptides-5–8 in spatial relation to the firefly luciferase active site. The cartoon representation shows the positions of peptides-5–8 relative to the active site. Peptide-5 (magenta, residues 277–289 in RE_Ph_ and 280–291 in GB_Av_), peptide-6 (purple, residues 307–323 in RE_Ph_ and 310–326 in GB_Av_), peptide-7 (green, residues 328–339 in RE_Ph_ and 331–342 in GB_Av_), and peptide-8 (orange, residues 346–370 in RE_Ph_ and 350–372 in GB_Av_) are highlighted in relation to AMP (cyan) and OxyLH_2_ (orange) in the active site. Peptides-5 and -6 are arranged approximately parallel to each other on one side of the active site, while peptides-7 and -8 are located on the opposite side, creating a defined structural framework around the catalytic cleft. The figure was generated using PyMOL Molecular Graphics System version 2.5.5 (Schrödinger, LLC).

Peptide-8 (residues 346–370 in RE_Ph_ and 350–372 in GB_Av_) is one of the longest peptides resolved in this study and comprises 25 and 23 residues, respectively (Fig 5). It includes the second strand of β-sheet-C, a long loop (∼20 residues), and the first strand of antiparallel β-sheet-D (Figs S7 and S8). Peptides-5/6 and -7/8 form two opposing walls of the active site cleft and are connected via backbone H-bonds (Fig S10). Notably, the H-bonding network between peptides-7 and -8 is more compact and stronger in GB_Av_ (2.7–3.1 Å) than in RE_Ph_ (3.5–4.4 Å). For instance, in GB_Av_, backbone atoms of I351 and T352 (peptide-8) form H-bonds with Q338 and Y340 (peptide-7), and the side chain of T352 additionally forms H-bonds (3.0 Å) with the backbone amine of Q338. These interactions are thought to reinforce the structural integrity of the ATP and luciferin binding pockets in GB_Av_. Similar interactions are present in RE_Ph_ but are weaker and more distant; importantly, an arginine insertion (R353) absent from all other beetle luciferases enlarges loop^351–364^ in RE_Ph_ and introduces greater conformational flexibility compared with loop^348–361^ of GB_Av_. This difference in structural connectivity correlates with HDX-MS data showing greater flexibility in RE_Ph_. In the apo state, WT and L334R RE_Ph_ and R337L GB_Av_ exhibited similar deuteration kinetics (∼50% → 70%) in peptide-8 upon incubation from 0.5 min to 3 h, whereas WT GB_Av_ showed significantly reduced dynamics (27% → 53%) (Figs 5C and 6E and F). The bound state reduced deuteration of peptide-8 across all variants to differing extents: WT GB_Av_ had the lowest exchange (∼20%) at all time points, suggesting a tightly stabilized active site cleft. Surprisingly, WT RE_Ph_ maintained a high degree of exchange in the bound state, comparable to the apo state of WT GB_Av_ (Fig 5C). These observations suggest that the active site of RE_Ph_ retains flexibility even when bound to substrate, potentially facilitating red-shifted emission by permitting the chromophore to adopt a more relaxed conformation. The flexibility is likely enhanced by the longer H-bond distances and R353 insertion in peptide-8.

Extensive mutagenesis studies have elucidated the pivotal roles of peptides-6–8 in regulating the bioluminescence color of beetle luciferases. Peptide-6 contains a highly conserved loop, S314–L319 (G_Pp_ numbering), that forms part of the luciferin-binding pocket (Fig S3) (Branchini et al, 2003). Two glycine residues in this loop are especially critical: the alanine substitutions G315A and G316A red-shift G_Pp_ emission from 557 to 607 nm and 578 nm, respectively, whereas also markedly reducing catalytic efficiency and substrate affinities (Branchini et al, 2003). These glycine residues appear essential for maintaining loop flexibility to enable conformational adjustments during catalysis and proper substrate alignment. Loss of flexibility leads to structural rearrangements that favor red-shifted emission but compromise enzymatic function. This illustrates a recurring theme: conformational plasticity around the active site facilitates lower energy photon emission, often at the cost of reduced catalytic efficiency.

Peptide-7 forms a key active site loop that shapes the luciferin and ATP binding pockets; its influence on emission is governed by conserved residues and interdomain interactions. A key example is R337 in GB_Av_, which forms a stabilizing salt bridge with E354 from the N-terminal domain (Viviani et al, 2016; Carrasco-López et al, 2018). This ionic contact stabilizes loop^351–364^, maintaining a closed, rigid active site that restricts solvent exposure and promotes green emission. Disruption of this salt bridge by the R337L mutation red-shifts GB_Av_ emission by 42 nm (538 → 580 nm) (Viviani et al, 2016; Carrasco-López et al, 2018). Conversely, L334 in RE_Ph_, the structural analog of R337, lacks this charge interaction, contributing to a more flexible and solvent-accessible active site. Introduction of L334R in RE_Ph_ partially restores rigidity, producing an 18-nm blue shift (623 → 605 nm), though not fully reversing the red-emission phenotype (Viviani et al, 2016; Carrasco-López et al, 2018). The functional importance of this region is conserved across luciferase families. In GL_Ms_, the mutations R337E and R337K red-shift emission from 573 nm to 602–605 nm, whereas in GL_Pt_, the R334A mutation red-shifts emission from 544 to 575 nm (Viviani et al, 2016; Carrasco-López et al, 2018). In addition, the T343A mutation of peptide-7 in G_Pp_ leads to a 60-nm red shift (558 → 617 nm), further implicating this flexible loop as a crucial determinant of color emission (Branchini et al, 2000; Modestova et al, 2014). These asymmetric effects highlight the environment-specific nature of emission tuning and show that greater rigidity does not necessarily reverse a red shift or restore blue-shifted emission.

Peptide-8 spans the back wall of the active site and anchors interactions between β-sheets C and D. It contains multiple residues that modulate emission by altering loop dynamics and solvent access. In G_Pp_, the I351A mutation shifts emission from 557 to 573 nm and reduces luciferin affinity, suggesting increased flexibility around the binding pocket (Branchini et al, 2003). In RE_Ph_, the N351K substitution—alone or in combination with I212L—blue-shifts the red emission from 630 nm to 621–620 nm (Li et al, 2010).

A defining difference between red- and green-emitting luciferases is the presence of R353 in RE_Ph_, a residue absent from all known green/yellow luciferases (Fig S3). Inserting an arginine at the equivalent position in other species consistently produces significant red shifts. For instance, R356 insertion in GL_Tu_ shifts emission by 61 nm (555 → 616 nm) (White et al, 1980), and R356 or K356 insertion in G_Pp_ produces 51-nm shifts (557 → 608 nm) (Moradi et al, 2009; Maghami et al, 2010). Even negatively charged insertions (E356) cause modest red shifts, likely by altering the local dielectric environment or displacing loops (Moradi et al, 2009; Maghami et al, 2010). Structural modeling suggests that these insertions may disrupt ionic contacts present in green-emitting luciferases, such as D356–K364, thereby promoting loop flexibility and increasing the hydration of the chromophore site (Carrasco-López et al, 2018). Interestingly, deletion of R353 from RE_Ph_ does not affect its red emission, suggesting that this residue alone does not determine color but may act synergistically within the RE_Ph_-specific structural context (Carrasco-López et al, 2018).

Another residue in this region, E354 (corresponding to N351 in RE_Ph_) (Fig S3), in green-emitting luciferases, further exemplifies how point mutations influence emission. Substituting E354 with R or K in GL_Tu_ produces bimodal spectra with red-shifted peaks at 606–610 nm (Nishiguchi et al, 2015). When combined with the R356 insertion, E354R or E354K results in a single, highly red-shifted peak at 602 nm. Conversely, N351K in RE_Ph_ modestly blue-shifts the emission from 630 to 621 nm (Branchini et al, 2000).

In summary, peptides-6–8 form a crucial interconnected network for fine-tuning beetle luciferase emission. Disrupting ionic contacts or increasing loop mobility, especially in the glycine-rich segment of peptide-6 or the salt bridge network of peptide-7, leads to red-shifted emission and, often, reduced activity. Conversely, restoring rigidity via charged substitutions or tighter H-bonding in peptide-8 results in blue shifts by constraining the active site. Subtle side chain changes in peptide-8 modulate the chromophore environment through local and propagated effects on H-bonding and loop stability, highlighting cooperative effects across peptides-7 and -8, where increased rigidity yields higher energy emission. Color tuning arises from distributed structural features, not single residues. The asymmetric effects of reciprocal mutations reveal that structural plasticity and rigidity are not simply reversible but depend on complex intramolecular interactions. This understanding can guide the rational engineering of luciferases with spectral and catalytic properties tailored for diverse applications.

### HDX-MS profiles of the C-terminal domain

**Figure S11.**
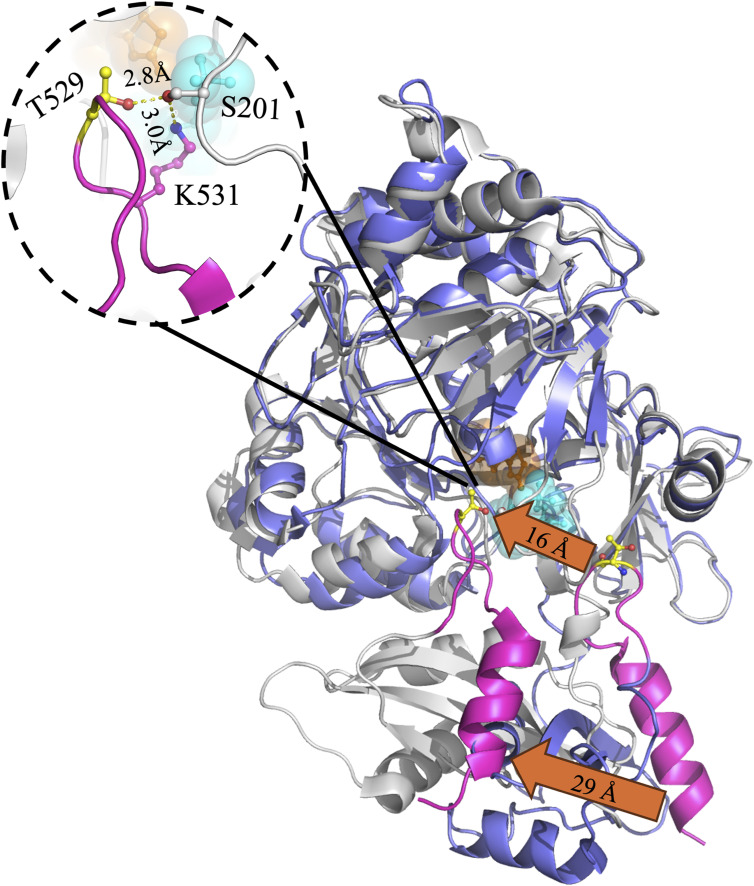
C-terminal domain movement during closure of the firefly luciferase active site. The cartoon representation shows the overlay of the open, apo state of GB_Av_ (PDB code: 6AAA, purple) with the closed, ligand-bound state of G_Pp_ (PDB code: 2D1R, white). OxyLH_2_ is shown in orange, and AMP is shown in cyan. Loop^523–530^ and the final α-helix-15 of the C-terminal domain are highlighted in pink to illustrate the large conformational change and 16–29 Å rotation between the open and closed states. **Inset**: In the closed conformation of G_Pp_, the side chains of T529 and K531 in the C-terminal domain form H-bonds at 2.8 and 3.0 Å, respectively, with the side chain of S201 in the N-terminal domain, thereby stabilizing the closed active site configuration. The figure was generated using PyMOL Molecular Graphics System version 2.5.5 (Schrödinger, LLC).

The C-terminal domain of beetle luciferases (residues 436–545) is characterized by a β-sheet core surrounded by three α-helices, H13–H15 (Fig S1). The β-sheet consists of three antiparallel β-strands; a β-turn connects the longest two strands. A conserved 13-residue loop^521–528^ RE_Ph_ or loop^523–530^ GB_Av_ links the third, shorter β-strand to the final α-helix-H15 of the C-terminal domain. Loop^523–530^ is conserved in all beetle luciferases and plays a key role in regulating the active site closure, color emission, thermodynamic stability, and catalytic activity (Nakatsu et al, 2006; Carrasco-López et al, 2018). Structural comparisons between the open state of GB_Av_ (PDB: 6AAA) and the closed state of G_Pp_ (PDB: 2D1R) reveal that loop^523–530^ undergoes large rotation and movement (16–29 Å) toward the N-terminal domain during active site closure (Fig S11). This transition facilitates new bonding interactions between the C- and N-terminal domains (Nakatsu et al, 2006; Carrasco-López et al, 2018). For example, the side chains of T292 and K526 in the N- and C-terminal domains form a 3.2 Å H-bond (Fig S11), whereas T529 and K531 of the C-terminal domain interact with S201 of the N-terminal domain (2.8 and 3.0 Å, respectively), reinforcing active site stabilization essential for catalysis. Thus, dynamic differences are expected between the apo and substrate-bound states of luciferases, with the bound state exhibiting reduced flexibility.

**Figure 7.**
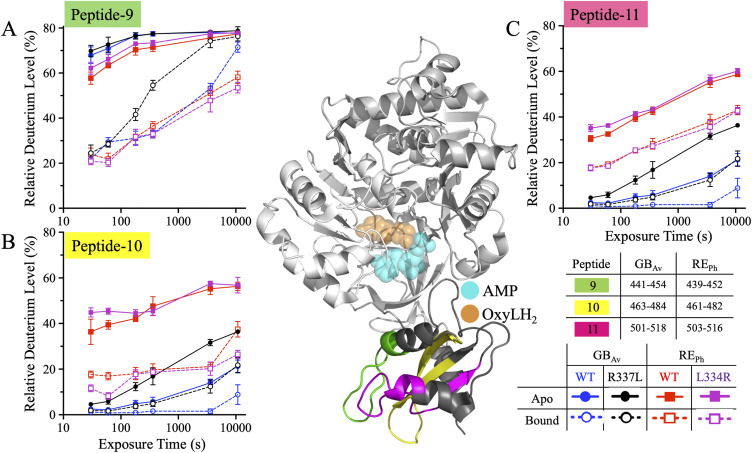
HDX-MS profiles of peptides-9–11 in the C-terminal domain of RE_Ph_ and GB_Av_. The cartoon representation depicts the monomeric GB_Av_ structure (PDB: 6AAA) as described in Fig 1. Peptides-9–11 are colored green, yellow, and pink, respectively. The format of the deuterium incorporation graphs is similar to those in Fig 1. **(A)** Peptide-9 exhibited high deuterium uptake in all enzyme variants in the apo state, ranging from ∼60% in RE_Ph_ to ∼70% in GB_Av_ at early time points. By 3.0 h, uptake reached ∼80% across all variants, including the bound state for GB_Av_ variants. In the bound state, deuterium uptake increased from an initial ∼20% to ∼55% for RE_Ph_ and ∼80% for GB_Av_ by 3.0 h. **(B)** Peptide-10 exhibited the highest deuterium uptake in the RE_Ph_ variants in the apo state. By contrast, WT GB_Av_ had lower uptake than R337L. A similar pattern was observed in the bound state, with RE_Ph_ variants showing higher uptake than the GB_Av_ variants and WT GB_Av_ incorporating more deuterium than R337L GB_Av_. **(C)** Peptide-11 exhibited a deuterium exchange profile similar to that of peptide-10, with the highest uptake observed in RE_Ph_ variants in the apo state. R337L GB_Av_ showed higher uptake than WT GB_Av_. In the bound state, RE_Ph_ variants retained the highest uptake, while WT GB_Av_ showed the lowest dynamics. The structural figure was generated using PyMOL Molecular Graphics System version 2.5.5 (Schrödinger LLC). The data are the mean ± S.D. of five to eight replicates.

**Figure 8.**
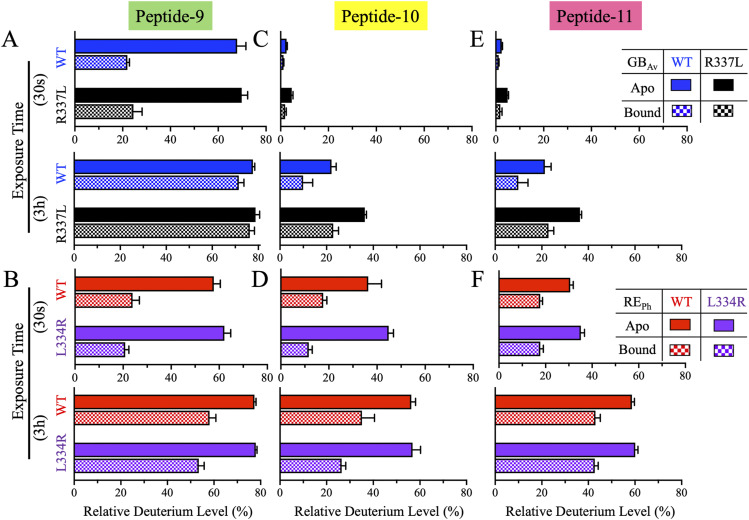
Bar plots of deuterium uptake by peptides-9–11 of RE_Ph_ and GB_Av_ at time points of 0.5 min and 3.0 h. The color coding and labels are similar to those in Fig 2. **(A, C, E)** Bar plots of relative deuterium incorporation by peptides-9, -10, and -11 of GB_Av_ at incubation time points of 0.5 min and 3.0 h. **(B, D, F)** Bar plots of relative deuterium incorporation by peptides-9, -10, and -11 of RE_Ph_ at incubation time points of 0.5 min and 3.0 h. The data are the mean ± S.D. of five to eight replicates.

Peptide-9 (residues 439–452 in RE_Ph_ and 441–454 in GB_Av_) lies at the beginning of the C-terminal domain and forms part of the flexible hinge region (residues 436–440) connecting the N- and C-terminal domains and the first turn of α-helix–H13 (Figs 7, S7, and S8). This region undergoes substantial conformational change (∼16–29 Å movement) during active site closure (Nakatsu et al, 2006; Carrasco-López et al, 2018). HDX-MS revealed that peptide-9 exhibited high deuterium uptake (>60%) in the apo state for all enzymes, with WT and R337L GB_Av_ showing slightly higher dynamics than WT and L334R RE_Ph_ (Fig 7A). In the apo state, the hinge region is fully exposed to solution, enabling high deuteration, with >60% deuterium labeling of peptide-9 at 0.5 min (Figs 8A and B). Upon substrate binding, deuterium uptake dropped significantly within the first 3 min (20–45%), confirming solvent shielding during active site closure. At longer time points, however, a gradual increase in deuteration was observed, likely reflecting the intrinsic flexibility of the hinge region connecting the C- and N-terminal domains. This hinge undergoes large-scale “breathing” motions even in the ligand-bound state, as supported by the pronounced conformational differences observed when comparing apo-crystal structures of RE_Ph_ and GB_Av_ with the closed, ligand-bound conformations of G_Lc_ (Nakatsu et al, 2006; Carrasco-López et al, 2018). Notably, the time-dependent deuterium profiles of the L334R and R337L mutants mirrored those of their WT counterparts, suggesting that active site closure dynamics mediated by the C-terminal domain and hinge region are largely conserved and are not related to color emission.

Studies of K443 in the β-hairpin motif of the C-terminal domain of the North American firefly luciferase G_Pp_ further support the functional importance of this region (Branchini et al, 2005b). A K443A mutation led to a ∼2,700-fold reduction in activity, underscoring the critical role of hinge flexibility for catalysis, independent of emission color.

Peptides-10 and -11 are both situated within the C-terminal domain and define a structurally cohesive unit associated with β-sheet-E. These peptides contribute to long-range allosteric regulation of luciferase emission properties. Peptide-10 (residues 461–482 in RE_Ph_ and 463–484 in GB_Av_) forms the two long antiparallel β-strands of the three-stranded β-sheet-E, whereas Peptide-11 (residues 503–516 in RE_Ph_ and 501–518 in GB_Av_) comprises α-helix-H14, a flexible connecting loop, and the final β-strand of β-sheet-E (Figs 7, S1, S7, and S8). In the apo state, deuterium uptake by these peptides at early time points was markedly greater in WT and L334R RE_Ph_ than in WT and R337L GB_Av_, consistent with a more flexible and solvent-exposed conformation. Peptide-10 reached ∼40% deuteration at 0.5 min in WT RE_Ph_, whereas deuteration in WT GB_Av_ was nearly static at ∼4% until 6 min (Figs 7B and 8C and D). Deuterium uptake by peptide-11 followed the same trend in the apo state; RE_Ph_ variants began with ∼35% deuteration, which increased to ∼60% at 3.0 h, whereas GB_Av_ variants started at ∼3% and reached 21% for WT GB_Av_ and 36% for R337L at 3.0 h (Figs 7C and 8E and F).

Importantly, R337L GB_Av_ consistently exhibited enhanced dynamics relative to WT GB_Av_ across both peptides and the apo and bound states. In peptide-10, R337L GB_Av_ showed ∼15% greater deuterium incorporation than WT GB_Av_ at 3.0 h (Figs 8C and D). A similar increase was observed in peptide-11, where R337L GB_Av_ reached 36% uptake compared with 21% in WT GB_Av_ by 3.0 h (Fig 8E). These effects were evident even in the bound state, in which R337L GB_Av_ consistently showed higher deuterium uptake than WT, indicating partial retention of flexibility upon substrate binding. Conversely, WT and L334R RE_Ph_ exhibited nearly identical deuteration kinetics across both peptides, indicating that the blue-shifting mutation does not significantly alter flexibility in this region. Substrate binding reduced overall exchange in all enzymes, but the relative order of dynamics persisted: R337L GB_Av_ > RE_Ph_ variants > WT GB_Av_. These consistent trends across structurally linked peptides reinforce the model that emission color correlates with conformational plasticity, suggesting that distal structural regions, such as β-sheet-E, contribute to global flexibility that shapes the chromophore environment.

Three key color-shifting mutations—F467S, E490V, and E490K—in the C-terminal domain of East European *Luciola mingrelica* luciferase (G_Lm_) have been identified as important determinants of bioluminescence emission color (Modestova & Ugarova, 2016). Both F467 and E490 are highly conserved solvent-exposed residues on the surface of the C-terminal domain, positioned far from the active site in the open conformation (Fig S3) (Viviani & Ohmiya, 2000). However, upon active site closure, they move toward the N-terminal domain at the interdomain interface, with E490 forming a 3.2-Å ionic interaction with the conserved residue K299. WT G_Lm_ displays pronounced pH-dependent shifts characterized by increasingly red-shifted emission at lower pH values, whereas E490V or E490K mutations induce a blue shift in the emission maximum at pH 7.0 from 576 to 564 or 566 nm, respectively, and abolish pH sensitivity. By contrast, F467S induces a pronounced red shift, increasing the WT emission at pH 7.0 from 576 to 610 nm, likely by altering local hydration and/or electrostatic conditions near the emitter to enhance the red-emitting component.

Several studies have underscored the pivotal role of E457, a highly conserved residue in the C-terminal domain, in determining the bioluminescence color of beetle luciferases (Koksharov & Ugarova, 2013; Modestova et al, 2014). Located at a key structural interface between the N- and C-terminal domains, E457 undergoes significant conformational rearrangement during active site closure, including a displacement of >10 Å and ∼120° rotation. This transition repositions E457 from a solvent-exposed surface to a component of the active site cover (Koksharov & Ugarova, 2013; Modestova et al, 2014). The E457K mutation induces a strong red shift from 566 to 604 nm at pH 7.8, whereas E457Q and E457V cause more moderate shifts to ∼574 nm (Koksharov & Ugarova, 2013; Modestova et al, 2014). These effects underscore the importance of the structural integrity of the C-terminal domain, which is maintained in part by an H-bond between the E457 side chain and the backbone amide nitrogen of V471 (3.0 Å), in preserving conformational requirements for specific color emission. Disrupting this interaction compromises active site architecture, altering the chromophore environment and emission properties.

In conclusion, Peptides 9–11 in the apo state are partially solvent-exposed and display notable conformational flexibility, consistent with their elevated deuterium uptake. Upon ligand binding, the C-terminal domain undergoes local rearrangements that enhance structural packing and establish additional stabilizing intermolecular contacts, thereby reducing solvent accessibility. This ligand-induced stabilization aligns with the lower deuterium uptake observed for Peptides 9–11 in the bound state and reflects their proximity to the active-site cleft in the ligand-bound luciferase structures.

**Figure S12.**
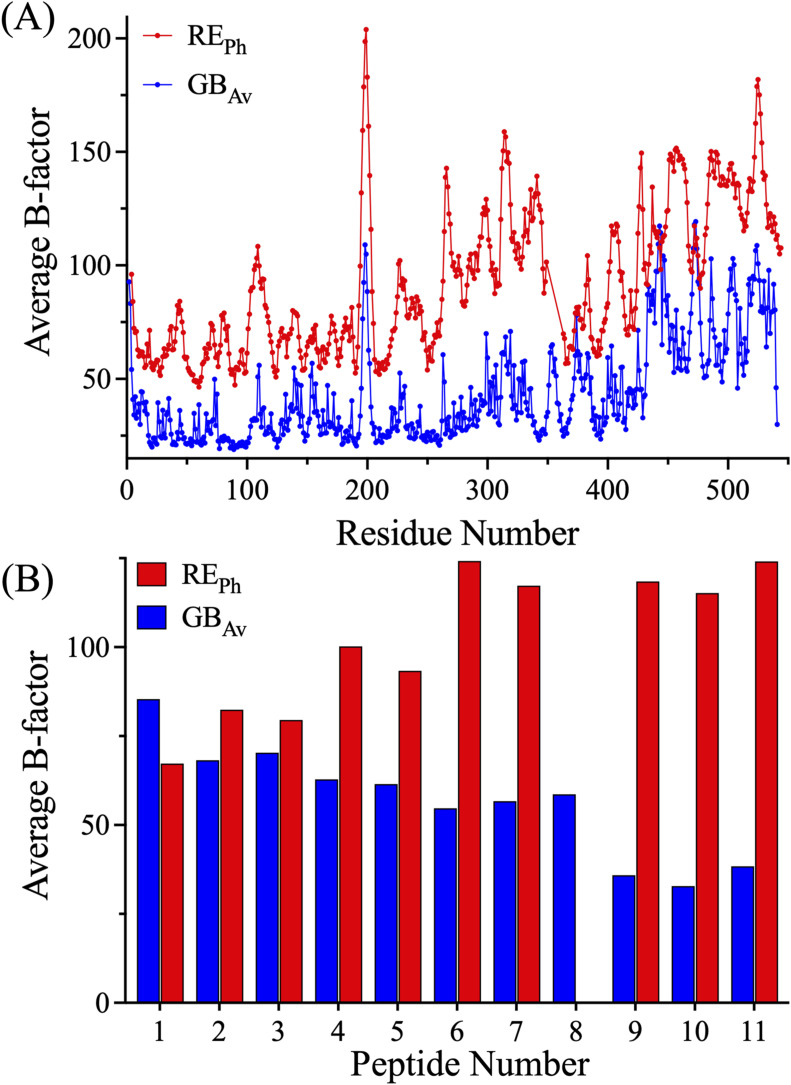
Crystallographic B-factor analysis of apo GB_Av_ and RE_Ph_ luciferases. **(A)** Residue-resolved crystallographic B-factors from the apo crystal structures of GB_Av_ (PDB: 6AAA) and RE_Ph_ (PDB: 6AC3) luciferases. RE_Ph_ displays globally higher B-factors, reflecting greater conformational flexibility compared with GB_Av_. **(B)** Bar plot of the average B-factors calculated for peptides 1–11 identified in the HDX-MS analysis. Consistently higher B-factors are observed for RE_Ph_ in most peptides. These results align with the HDX-MS data and support the conclusion that red emission correlates with enhanced local and global flexibility.

This work demonstrates that the color diversity of beetle luciferases—despite their use of identical substrates—arises from distributed, state-dependent protein dynamics. Our HDX-MS analyses reveal that red-emitting enzymes, exemplified by RE_Ph_ and the R337L GB_Av_ mutant, exhibit greater global flexibility than their more rigid, blue-green-emitting counterparts, such as GB_Av_ and the L334R RE_Ph_ mutant. This relationship is independently supported by crystallographic B-factors, confirming the intrinsic flexibility of RE_Ph_ (Fig S12). High-resolution mapping identifies crucial dynamic “hotspots” flanking the active site, where increased mobility promotes a relaxed chromophore environment that favors red-shifted emission. Key regions within the N-terminal domain—including loop^223–235^ (Peptide-2), the glycine-rich segment (Peptide-6), and the salt-bridge/loop network (Peptides-7/8)—undergo destabilization through mechanisms such as the loss of the R337–E354 salt bridge or the R353 insertion. This inherent flexibility, amplified by a weakened hydrophobic core in β-sheet-A (Peptides-4/5), propagates allosterically, leading to enhanced dynamics in the C-terminal β-sheet-E (Peptides-10/11). In contrast, the structural rigidity and tight packing of GB_Av_ stabilize a higher-energy blue-green light emission. The limited spectral blue-shift achieved by the L334R mutation in RE_Ph_ underscores an inherent asymmetry in dynamically engineering color control. Collectively, these results establish a coherent, dynamics-centric framework that explains decades of mutational data and positions conformational plasticity as a principal determinant of emission color, providing a robust foundation for rationally engineering luciferases with tailored spectral properties for advanced imaging and sensing applications.

## Materials and Methods

### Expression and purification of beetle luciferases

The genes encoding recombinant WT and mutant *GB*_*Av*_ and *RE*_*Ph*_ were introduced into the pET28b bacterial expression vector by GenScript Inc. N-terminal Hisx6-tagged luciferase proteins were expressed in *E. coli* BL21-CodonPlus-RIL (Stratagene). The inoculated cultures (4–6 liter) were grown in LB supplemented with 100 mg/liter kanamycin and 50 mg/liter chloramphenicol at 25°C until the OD_600_ reached 0.2. The temperature was then lowered to 15°C, and expression was induced overnight with 0.1 mM IPTG. The cells were harvested by centrifugation at 12,000*g* at 4°C for 10 min in an Avanti J26-XPI centrifuge (Beckman Coulter Inc.) and resuspended in lysis buffer (100 mM Tris pH 7.5, 150 mM NaCl, 5 mM imidazole, 3 mM βME, and 0.1% protease inhibitor cocktail [P8849; Sigma-Aldrich]). The cells were lysed by sonication on ice and then centrifuged at 40,000*g* for 45 min at 4°C. The supernatant was loaded onto ProBond Nickel-Chelating Resin (Life Technologies) previously equilibrated with binding buffer (100 mM Tris pH 7.5, 150 mM NaCl, 5 mM imidazole, and 3 mM βME) at 4°C. The resin was washed with 10 column volumes (cv) of binding buffer, followed by 15 cv of washing buffer (100 mM Tris pH 7.5, 150 mM NaCl, 25 mM imidazole, and 3 mM βME). The His-tagged luciferase enzymes were eluted from the Ni-column with 100 mM Tris, pH 7.5, 150 mM NaCl, 300 mM imidazole, and 3 mM βME in 1-ml aliquots. Finally, fractions containing luciferase enzymes were loaded onto a HiLoad Superdex-200 size-exclusion column pre-equilibrated with 50 mM Hepes pH 7.5, 150 mM NaCl, and 0.5 mM TCEP on an AKTA purifier core system (GE Healthcare). The final protein samples were collected and concentrated to ∼100 μM based on the Bradford assay, and their purity was assessed by SDS–PAGE.

### Bioluminescence emission kinetics

Bioluminescence emission spectra were acquired on a Cytation 5 multi-mode microplate reader (BioTek Instruments). Reactions contained 20 mM Hepes, pH 7.5, 150 mM NaCl, 20 mM MgCl_2_, 0.5 mM TCEP, 2 mM luciferin, 2 mM ATP, and 2 μM enzyme. Spectra were scanned from 450 to 700 nm in 2 nm increments and recorded at 4 min intervals to track emission decay over time. The Detector gain and integration time were fixed for all measurements, with settings pre-validated to avoid saturation, enabling direct comparison across enzyme variants. For kinetic summaries, time-decay profiles at each enzyme’s *λ*_max_ were extracted to quantify the decrease in luminescence intensity under saturating substrate conditions.

### Hydrogen/deuterium exchange (HDX) deuterium labeling of beetle luciferases

The HDX reaction for deuterium labeling of WT and mutant beetle luciferase enzymes was performed in an HDX PAL RTC autosampler (LEAP Technologies) with temperature-controlled chambers. The experiments were initiated by incubating 3 μl of 100 μM luciferase variants in 37 μl of 99.9% D_2_O (Sigma-Aldrich) buffer (20 mM Hepes pD 7.5, 150 mM NaCl, 50 mM MgCl_2_, and 0.5 mM TCEP) at 25°C for 0.5, 1.0, 3.0, 6.0 min, 1, or 3 h. Under these conditions, the maximum achievable deuterium concentration in the labeling mixtures was 92.5%. Deuterium exchange measurements were repeated five to eight times, including both technical and biological replicates, in the presence or absence of 5 mM luciferin and 5 mM ATP. Replicates with poor or unreliable MS signals were excluded from analysis. The final number of replicates included in HDX calculations varied (five to eight per time point) depending on data quality. Reported results represent the mean values across replicates, with standard deviations used to assess measurement precision and shown as error bars. The exchange reaction was quenched at 4°C by 1:1 dilution (vol/vol) in quench buffer (2 M guanidine-HCl, 100 mM phosphate buffer pH 2.2, 50 mM MgCl_2,_ and 200 mM TCEP). For protein sequence identification, a non-deuterated run was performed under the same conditions and using the same quench buffer.

For in-line enzyme digestion and analysis, an isocratic pump (MX-Class; Teledyne SSI) was used to load 70 μl of the final quenched HDX mixture onto an immobilized protease type XIII/pepsin column (w/w 1:1; NovaBioAssays LLC) at 4°C and a flow rate of 100 μl/min in 0.3% formic acid. The digested sample was then injected into a C18 Trap column (1.7 μm × 30 mm; Waters) followed by an analytical C18 column (1.7 μm × 100 mm; Waters) at 4°C on a liquid chromatography system (1290 Infinity; Agilent Technologies) to desalt and separate the generated peptides. The peptides were separated by applying a 10-min gradient from 5% to 40% acetonitrile in 0.3% formic acid at a constant flow rate of 40 μl/min, which was followed by 95% acetonitrile for 1 min. A 10 min wash with 95% acetonitrile in 0.3% formic acid was used to prevent peptide carryover to the next run. Samples for sequence mapping and total peptide coverage based on tandem mass spectrometry (MS/MS) were performed similarly, except that D_2_O was replaced with H_2_O in the labeling phase. The LC system was coupled to a QTOF Impact II mass spectrometer (Bruker Daltonik) equipped with an Easy Spray ion source and operated in positive ion mode. The spray voltage was 4.5 kV with dry gas of 4.0 liters/min and dry temperature of 100°C, and full scans were acquired in a TOF mass analyzer over m/z 300–2,000 at a spectrum acquisition rate of 2.0 Hz. Sequence mapping and peptide identification based on auto MS/MS with a fixed precursor cycle of 2 s were performed for non-deuterated samples.

### Data processing

For sequence mapping, MS/MS raw files with *m/z* 622 lock mass calibration were converted to mgf format by DataAnalysis software (Bruker Daltonik). Searches against the luciferase variant sequences were performed using ProteinScape software (Bruker Daltonik) with an in-house Mascot search engine (Matrix Science Limited). The search parameters were set to peptide tolerance of 10 ppm, MS/MS tolerance of 0.05 D, and mascot score of 20 for positive peptide identification. Oxidation of methionine and N-terminal acetylation were used as variable modifications.

HDExaminer software (Sierra Analytics, version 2.5.1, 64-bit) was used to calculate the percentage deuterium uptake (%D) for all protease-generated peptides, based on peptide size and the theoretical maximum deuterium incorporation. Peptides not detected in the WT enzyme were excluded from mutant analysis. HDExaminer automatically isolates isotopic envelopes and calculates the average mass of both deuterated and non-deuterated peptides. For each detected peptide, the %D and SD were calculated at each incubation time point (0.5, 1.0, 3.0, 6.0 min, 1, and 3 h), with a minimum of five to eight replicates per time point. Measurements were excluded if deuterium uptake could not be calculated or if the SD exceeded 5%. Differences in deuterium uptake were considered significant if consistently greater than 4%. Peptide mapping and structural visualization were performed using PyMOL (Schrödinger), and further data analysis was conducted using GraphPad Prism version 10.3.1 for macOS (GraphPad Software).

## Supplementary Material

Reviewer comments

## Data Availability

The authors declare that all data that support the findings of this study are available within the paper files and as supplementary documents.
